# Synthesis, in vitro, and in vivo evaluation of novel *N*-phenylindazolyl diarylureas as potential anti-cancer agents

**DOI:** 10.1038/s41598-020-74572-1

**Published:** 2020-10-21

**Authors:** Lucas N. Solano, Grady L. Nelson, Conor T. Ronayne, Shirisha Jonnalagadda, Sravan K. Jonnalagadda, Kaija Kottke, Robert Chitren, Joseph L. Johnson, Manoj K. Pandey, Subash C. Jonnalagadda, Venkatram R. Mereddy

**Affiliations:** 1 Integrated Biosciences Graduate Program, University of Minnesota, Duluth, MN 55812 USA; 2 Department of Chemistry and Biochemistry, University of Minnesota Duluth, Duluth, MN 55812 USA; 3 Department of Chemistry and Biochemistry, Rowan University, Glassboro, NJ 08028 USA; 4 Department of Biomedical Sciences, Cooper Medical School of Rowan University, Camden, NJ 08103 USA; 5 Department of Pharmacy Practice and Pharmaceutical Sciences, University of Minnesota, Duluth, MN 55812 USA

**Keywords:** Cancer therapy, Medicinal chemistry, Drug discovery and development, Cancer

## Abstract

Novel *N*-phenylindazole based diarylureas have been designed, synthesized and evaluated as potential anticancer agents. In vitro cell viability studies of these derivatives illustrate good potency with IC_50_ values in the range of 0.4–50 μM in several cancer cell lines including murine metastatic breast cancer 4T1, murine glioblastoma GL261, human triple negative breast cancer MDA-MB-231, human pancreatic cancer MIAPaCa-2, and human colorectal cancer cell line WiDr. The ester group in the lead compound **8i** was modified to incorporate amino-amides to increase solubility and stability while retaining biological activity. Further in vitro studies reveal that lead candidates inhibit tube length in HUVEC cells. In vivo systemic toxicity studies indicate that these candidate compounds are well tolerated in mice without any significant side effects. Anticancer efficacy studies in WiDr tumor xenograft and 4T1 tumor syngraft models demonstrate that the lead candidate **11** exhibits significant antitumor properties as a single agent in these tumor models.

## Introduction

Nitrogen‐containing heterocycles are pharmacologically important scaffolds and are present in numerous clinically approved drugs^1–3^. Nitrogenous heterocycles offer a unique opportunity in drug development as they readily allow for the manipulation of lipophilicity, polarity, and hydrogen bonding^1–3^. These manipulations could potentially result in improved physiochemical properties including pharmacokinetics, pharmacodynamics, and toxicological profile to enhance efficacy of drugs^1–3^. Indazoles are a class of nitrogen-containing heterocycles related to the naturally found indoles, are comprised of a benzene fused to a pyrazole and can exist as 1*H*‐indazole, 2*H*‐indazole, and 3*H*‐indazole. Indazoles have been extensively studied for their pharmaceutical properties and have been shown to have high compatibility for drug development^4–6^. Literature reports indicate that the indazole structural unit is utilized in numerous pharmacologically active agents including drugs with anti-cancer properties^4–6^. For example, indazole-based pazopanib and axitinib have been developed as multi-tyrosine kinase inhibitors that are clinically indicated for the treatment of a variety of cancers^7–16^.

Ureas are carbonyl groups flanked by two nitrogens which have unique hydrogen bonding capabilities and as a result, they are frequently included in the drug design and discovery process^17^. Diaryl ureas have recently been explored as pharmacophores for the development of anticancer agents with numerous mechanisms of action, including anti-angiogenesis^17^. Examples of diaryl urea containing anticancer agents include regorafenib and sorafenib with clinical indications for colorectal and kidney cancers, respectively^18–23^.

**Figure 1:**
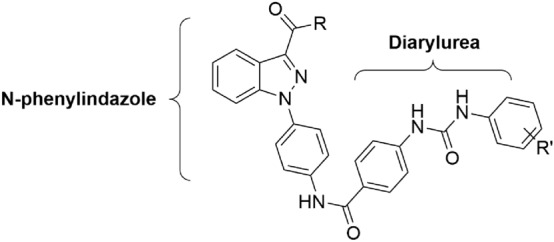
Structural design of *N*-phenylindazolyl diarylureas as potential anticancer agents.

Owing to the importance of indazoles and diaryl ureas in drug development, we envisioned that conjugating indazole template with diphenyl urea would provide an opportunity to design novel drug candidates with angiogenesis inhibition properties (Fig. 1). In the current work, we have synthesized and evaluated a series of *N*-phenylindazolyl diarylureas derivatives as potential anticancer agents.

## Results

**Figure 2:**
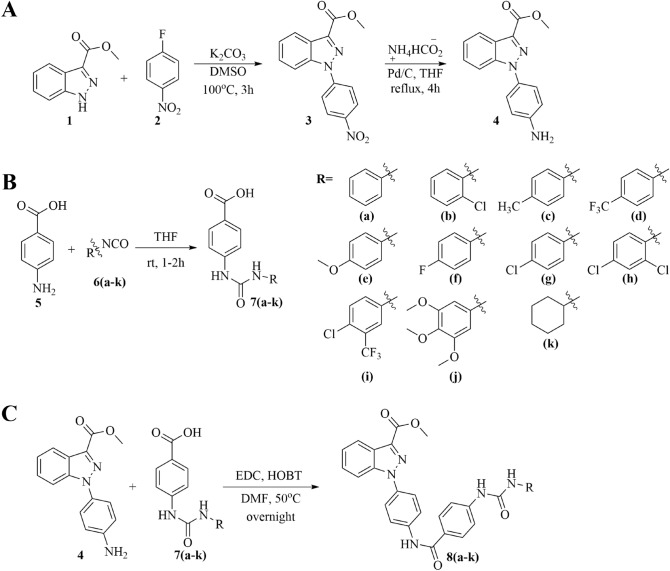
(**A**) Synthesis of methyl 1-(4-aminophenyl)-1H-indazole-3-carboxylate for use in coupling with synthesized (**B**) para-ureido carboxylic acids **7a–7k**. (**C**) Synthesis of *N*-phenylindazole diarylureas **8a–k**.

Synthesis of *N*-phenylindazolyl diarylureas was initiated by utilizing commercially available starting material methyl 1H-indazole-3-carboxylate **1**, which was first treated with 4-fluoronitrobenzene **2**. The reaction underwent substitution under basic conditions to provide the corresponding methyl 1-(4-nitrophenyl)-1H-indazole-3-carboxylate **3**. The nitro group in **3** was reduced in the presence of Pd/C and ammonium formate to the corresponding amine **4** (Fig. 2A). Separately, *p*-aminobenzoic acid **5** was reacted with various isocyanates **6a–k** to obtain the corresponding 4-(3-phenylureido)benzoic acids **7a–k** (Fig. 2B). Condensation of amine **4** under peptide coupling conditions with diphenylurea benzoic acids **7a–k** provided the corresponding indazole diphenylureas **8a–k** (Fig. 2C). Detailed synthetic protocols and spectral characterization can be found in the supplemental information.

**Figure 3:**
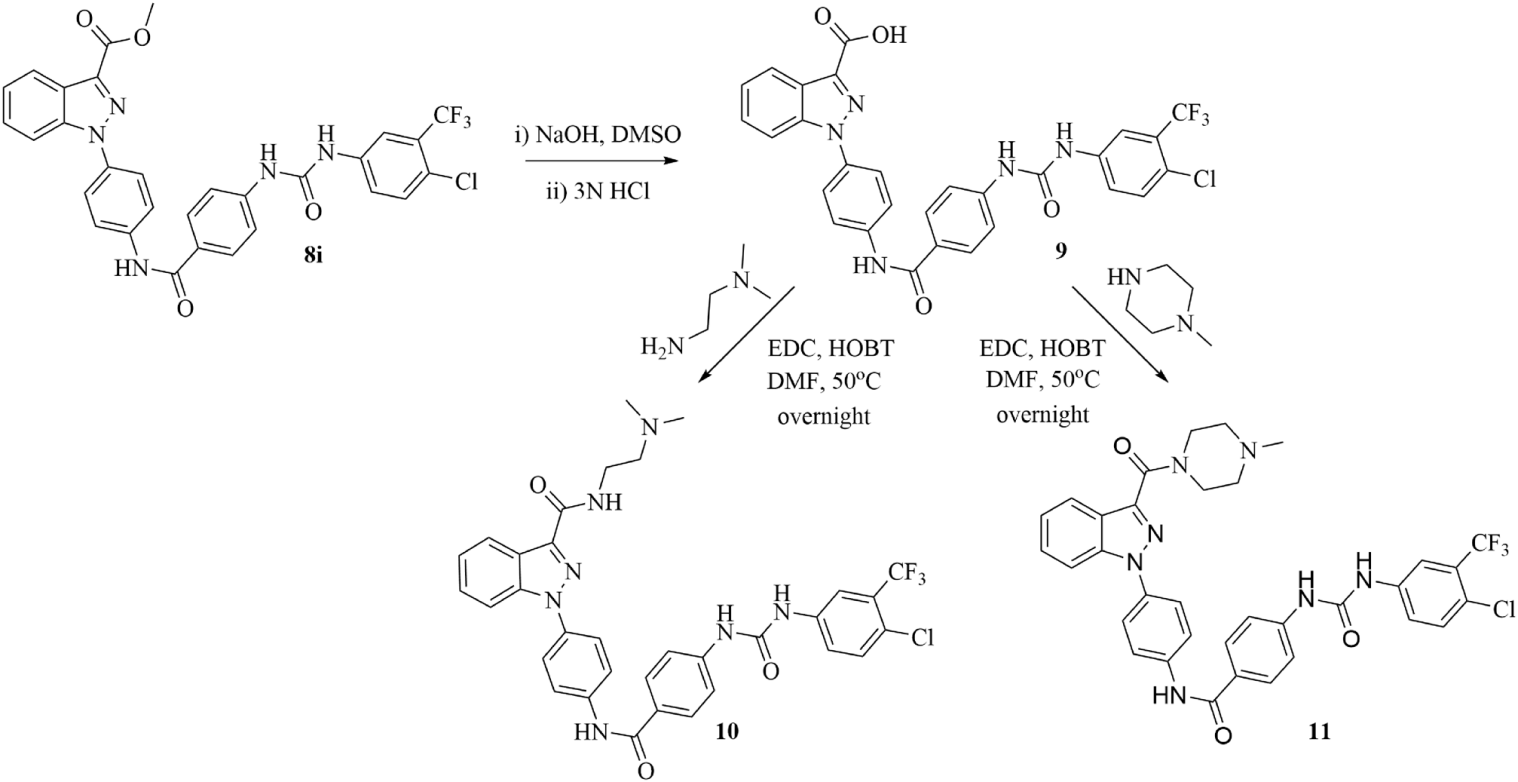
Hydrolysis of **8i** to generate acid **9** and corresponding amido-amines **10** and **11** with increased solubility and potential for enhanced stability.

**Figure 4:**
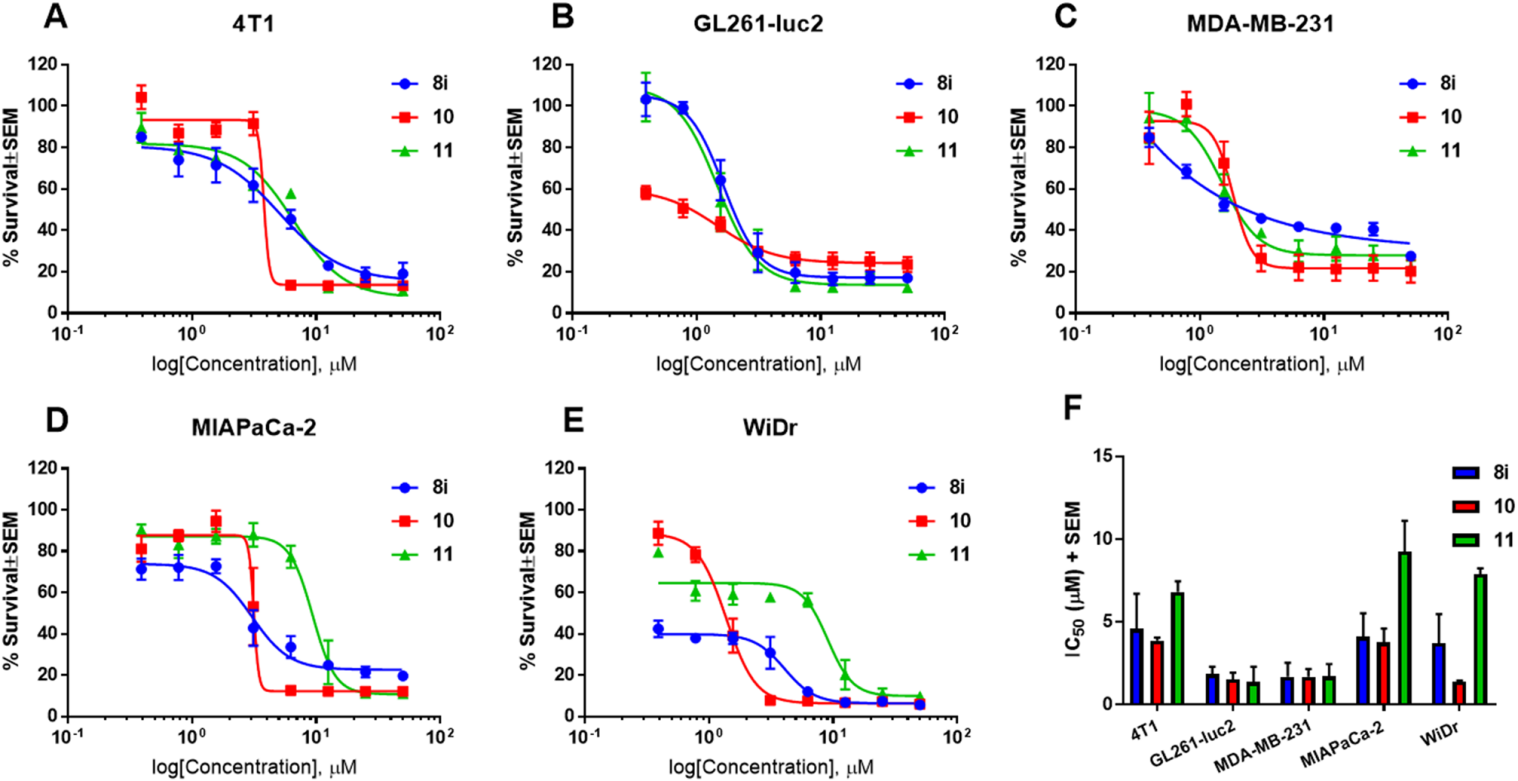
Graphical representation of in vitro cell viability assay dose–response curves of lead candidate compounds **8i**, **10**, and **11** in (**A**) 4T1, (**B**) GL261-luc2, (**C**) MDA-MB-231, (**D**) MIAPaCa-2, and (**E**) WiDr cell lines. (**F**) Note retained biological activity between parent ester compound **8i** and amido derivatives **10** and **11**. Error bars in (**A**–**E**) represent the SEM of six technical replicates across three independent experiments, with each concentration tested in duplicate for each experiment (n = 6). Error bars in (**F**)represent the SEM of the IC_50_ obtained from the three independent experiments (n = 3), and are derived from values in Table 1. All compounds were tested at the same concentration range (50–0.39 µM) for relative comparison.

*N*-phenylindazole diarylureas **8a–k** were then evaluated for their effects on cell viability utilizing 3-(4,5-dimethylthiazol-2-yl)-2,5-diphenyltetrazolium bromide (MTT) assay on aggressive and difficult to treat cancer cell lines including murine metastatic breast cancer 4T1, murine glioma GL261-luc2, human triple negative breast cancer MDA-MB-231, human pancreatic cancer MIAPaCa-2, and human colorectal adenocarcinoma WiDr. These cell lines are all derived from aggressive solid tumors, wherein angiogenesis may play an important role in both tumor growth and metastasis. Hence, these cell lines offer an appropriate platform to screen candidates containing anti-angiogenesis pharmacophores, including diarylurea template found in **8a–k**. Further, these cell lines represent both human and murine neoplasms; suitable for in vivo models in both clinically relevant xenograft and syngraft based anticancer efficacy studies. These studies indicated that compounds **8a–k** showed effects on cell viability in various cancer cell lines with IC_50_ values in the range of 0.7–50 µM concentration (Table 1). However, due to their poor solubility, we hydrolyzed the ester of one of the lead compounds **8i** into the corresponding carboxylic acid **9** (Fig. 3). Compound **8i** was chosen based on its superior effects on cell viability, and presence of metabolically stable 4-Cl, 3-CF_3_ phenylurea template^18–23^. However, the acid derivative **9** did not affect cell viability up to 50 µM concentration (Table 1), and we attributed the lack of biological activity to decreased cell membrane permeability of more polar carboxylate group. Further, we envisioned that the lipophilic methyl ester in **8i**, although biologically active in vitro, would be metabolically vulnerable to hydrolysis via plasma esterase activity in in vivo systems, and could result in pharmacologically inactive carboxylic acid **9**. In this regard, we sought to modify the ester group in **8i** by introducing stable and solubility-enhancing secondary and tertiary amido-amine units with retained lipophilic characteristics. We hydrolyzed **8i** and coupled the resulting carboxylic acid **9** with *N,N*-dimethylethane-1,2-diamine and 1-methyl piperazine to obtain the corresponding amido-amine derivatives **10** and **11**, respectively (Fig. 3). Gratifyingly, compounds **10** and **11** retained similar biological activity with regard to cell viability when compared to parent compound **8i** (Table 1, Fig. 4).

**Table 1:** MTT IC_50_ (µM) values of compounds **8a–k** and **9–11** in 4T1, GL261-luc2, MDA-MB-231 MIAPaCa-2, and WiDr cell lines.

Compound	4T1	GL261-luc2	MDA-MB-231	MIAPaCa-2	WiDr
**8a**	1.3 ± 0.4	1.9 ± 0.4	4.8 ± 2.9	14.3 ± 6.8	0.7 ± 0.3
**8b**	1.5 ± 0.3	1.5 ± 0.4	1.6 ± 0.2	20.7 ± 3.1	2.2 ± 0.8
**8c**	0.9 ± 0.3	1.4 ± 0.1	1.1 ± 0.2	11.1 ± 5.2	6.7 ± 0.1
**8d**	2.3 ± 0.5	3.9 ± 0.5	2.5 ± 0.5	4.7 ± 0.9	5.4 ± 1.1
**8e**	> 50	> 50	> 50	> 50	> 50
**8f**	3.0 ± 1.0	3.2 ± 1.2	4.6 ± 1.0	5.6 ± 0.6	2.0 ± 0.7
**8g**	1.9 ± 0.5	1.8 ± 0.1	5.7 ± 0.8	4.5 ± 0.5	2.7 ± 0.5
**8h**	1.5 ± 0.2	1.8 ± 0.4	2.4 ± 0.3	6.1 ± 0.4	4.0 ± 1.4
**8i**	4.6 ± 2.1	1.8 ± 0.4	1.7 ± 0.8	4.1 ± 1.4	3.7 ± 1.8
**8j**	> 50	> 50	> 50	> 50	> 50
**8k**	6.1 ± 0.3	4.3 ± 0.5	31.0 ± 6.5	5.6 ± 0.5	0.7 ± 0.1
**9**	> 50	> 50	> 50	> 50	> 50
**10**	3.9 ± 0.2	1.5 ± 0.4	1.7 ± 0.5	3.8 ± 0.8	1.4 ± 0.1
**11**	6.8 ± 0.6	1.4 ± 0.9	1.7 ± 0.8	9.2 ± 1.9	7.9 ± 0.3

Average ± SEM of a minimum three separate experiments.

**Figure 5:**
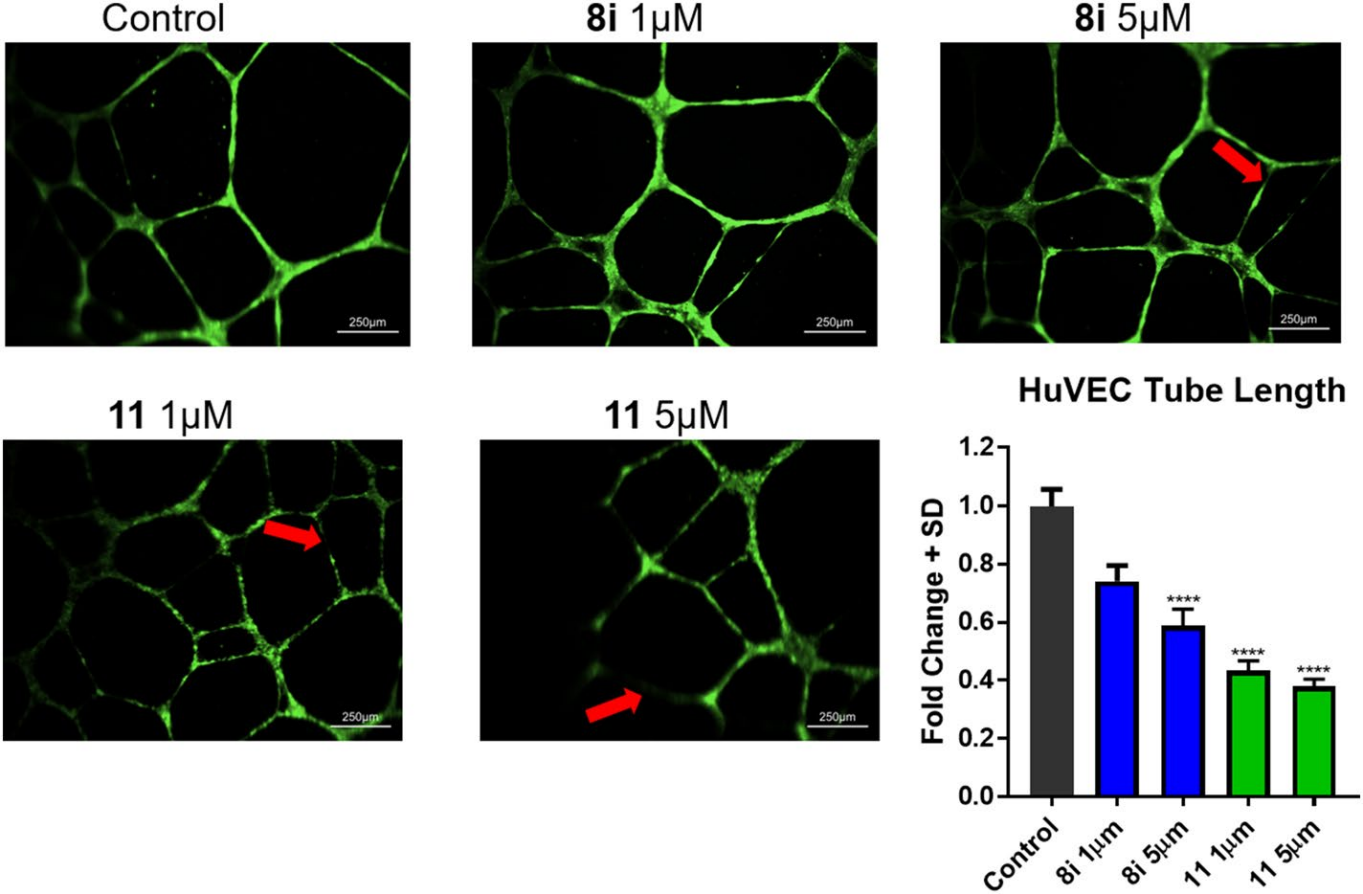
Compounds **8i** and **11** decrease HUVEC tube length in vitro. In vitro tube forming assay, and quantification of tube length was performed as described under “Materials and methods”. Briefly, HUVEC cells were seeded on top of matrigel and incubated for 4 h to form tubes following VEGF stimulation. Cells were then treated with 1 µM and 5 µM of compound **8i** and **11** for 12 h. The fold changes were calculated by comparing the tube length of treated versus non-treated cells. Statistical significance (Kruskal–Wallis one-way ANOVA) of tube length inhibition by compound **8i** and **11** was compared to the non-treated cells. All images were captured using the same magnification and are representative of 10 fields of view (see scale bar, 250 µm). Bars indicate Mean + SD of 30 independent tube length measurements (n = 30, ****P < 0.0001).

Based on their superior biological activity, we selected parent compound **8i** and metabolically stable analog **11** as lead candidates for preliminary in vitro mechanism of action studies. Amino-amide analog **10** also exhibited similar potency in vitro, but unhindered secondary amide may limit the translational potential when compared to the sterically hindered and stable tertiary amide of **11** with regards to esterase/amidase hydrolysis under physiological conditions. Hence, **10** was not subjected for further mechanism studies. Based on the track record of 1-(4-chloro-3-(trifluoromethyl)phenyl)-3-phenylurea template in providing anti-angiogenesis properties^21,22^, the lead candidates were screened for their ability to inhibit tube length in human umbilical vein endothelial cell line HUVEC. Tube forming ability of endothelial cell lines is a widely utilized method to evaluate angiogenesis^24^ and hence, compounds **8i** and **11** were screened for their ability to inhibit the tube formation of HUVEC in vitro. We treated HUVEC cells with increasing doses of **8i** and **11**, once initiation of tube formation took place. Total tube lengths were measured using Image J software and inhibitory response of compounds was estimated. These experiments indicated that candidate compound **8i** (5 µM) and more potently compound **11** (1 and 5 µM) inhibited HUVEC tube length in HUVEC cell lines (Fig. 5). Interestingly, at this concentration both compounds were not toxic to HUVEC cells (IC_50_ > 25 µM, 12 h; data not shown). Furthermore, we investigated the cytotoxic response of compound **8i** and **11** on HUVEC cells for 24 h, we noted similar response (IC_50_ > 25 µM, 24 h; data not shown). Thus, these compounds exhibited inhibitory properties at sub-toxic doses, suggesting the effects on tube length were not due to the cytotoxic response of these compounds.

**Figure 6:**
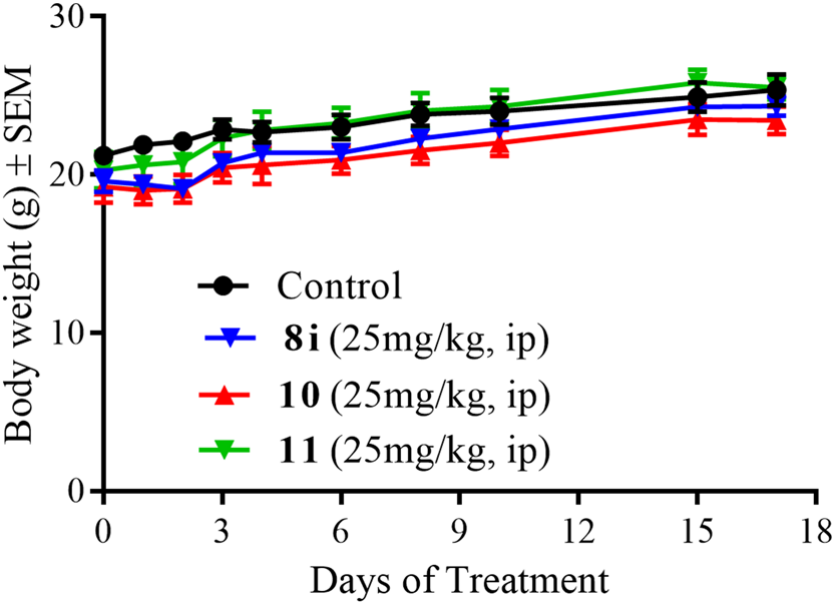
Systemic toxicity study in CD-1 mice. Mean body weight changes ± SEM (n = 6) in mice with the once daily intraperitoneal treatment of compounds **8i**, **10**, and **11**.

To understand the translational potential and general tolerance of these candidate compounds, in vivo systemic toxicity and efficacy studies were performed. For these studies, compounds **8i**, **10**, and **11** were chosen based on their superior in vitro potency in the experiments described above. Further, these compounds were also chosen to evaluate potential effects on in vivo efficacy of varying esterase/amidase vulnerabilities of ester (**8i**), secondary amide (**10**), and tertiary amide (**11**). Compounds **8i**, **10**, and **11** were administered once daily, intraperitoneally (i.p.) at a dosage of 25 mg/Kg for 17 days. Body weights, along with behavior, activity, and grooming patterns, were measured and observed every day initially, and later at ~ 2–5 days. At the end of the study mice in treatment groups showed no significant body weight loss and exhibited normal behavior, activity, and grooming patterns when compared to the control group (Fig. 6). Based on these studies, we deduced that the candidate compounds are in general well tolerated. However, organ function tests such as liver and kidney, along with necropsy studies of organ harvest and immunohistochemical analysis of microscopic changes need to be carried out to fully understand the systemic toxicity of these compounds.

**Figure 7:**
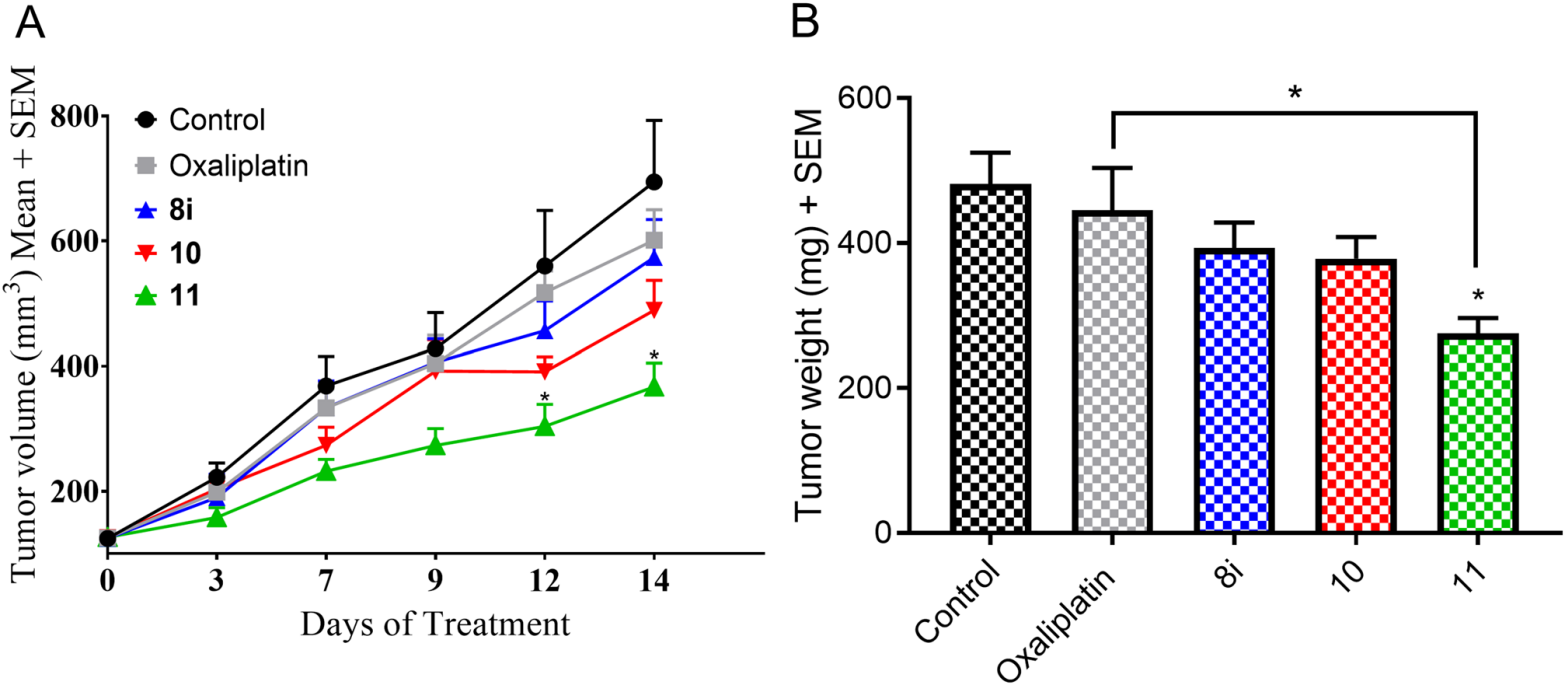
Tumor growth inhibition properties of lead compounds **8i**, **10**, and **11** in WiDr tumor xenograft model. Data represents the mean + SEM (n = 6) of (**A**) tumor volumes per day of treatment and (**B**) end-point tumor weights. Two-factor anova statistical analysis was utilized to compare tumor growth between treated groups, vehicle control, and positive control group oxaliplatin (*P < 0.05).

As the lead compounds **8i**, **10**, and **11** were well tolerated in mice, we next evaluated these compounds for anticancer efficacy on a colorectal cancer WiDr xenograft model. Colorectal cancer is the third most prevalent malignancy in terms of new cases and deaths in both men and women and hence, we sought to investigate the efficacy of candidate compounds in this tumor model. Athymic nude mice were injected with 5 × 10^6^ WiDr cells (suspended in 1:1 mixture of PBS-matrigel) subcutaneously on right flank. Tumors were measured every 2–3 days and treatment was initiated after tumors reached a mean volume of ~ 150 mm^3^. At this stage, mice were randomly assigned into groups (n = 6 mice per group) and mice in groups 1–3 were treated with **8i**, **10**, and **11**, respectively, at a dosage of 25 mg/kg, i.p, once daily, for 14 days. Group 4 was administered with clinically utilized colorectal cancer drug oxaliplatin at a dosage of 10 mg/kg, i.p. once daily and group 5 was injected with vehicle (10% DMSO, 10% PEG, 40% HS-15 (18.8% w/v in H_2_O), and 40% H_2_O). Tumor volume was recorded every 2–3 days, and starting on day-12, compound **11** showed statistically significant reduction of tumor with (*P* < 0.05) compared to control group (Fig. 7A). Based on the tumor volume, compound **11** exhibited 47% and 39% tumor growth inhibition compared to control and oxaliplatin treatment groups, respectively. At the end of the study, mice were euthanized, and the tumors were resected and weighed. In this case also, compound **11** showed significant reduction of tumor (*P* < 0.05) compared to the control group, which accounted for 43% and 38% of tumor growth inhibition compared control and oxaliplatin treatment groups, respectively (Fig. 7B). In support of our working hypothesis regarding the in vivo lability of methyl ester, we did not see any significant reduction of tumors with compounds **8i**. The *N,N*-dimethyl ethylene amide **10** exhibited slightly enhanced tumor growth inhibition properties, but lack of statistical significant efficacy may be attributed to insufficient metabolic stability of secondary amide when compared to the tertiary piperazine amide **11**. Additionally, for the duration of the experiment, body weight changes were observed in a similar fashion to that of systemic toxicity study (Fig. 6) to assess general tolerability of candidate compounds in tumor bearing mice. We observed normal body weight gains indicating that compounds were well tolerated in a treatment setting (data not shown).

**Figure 8:**
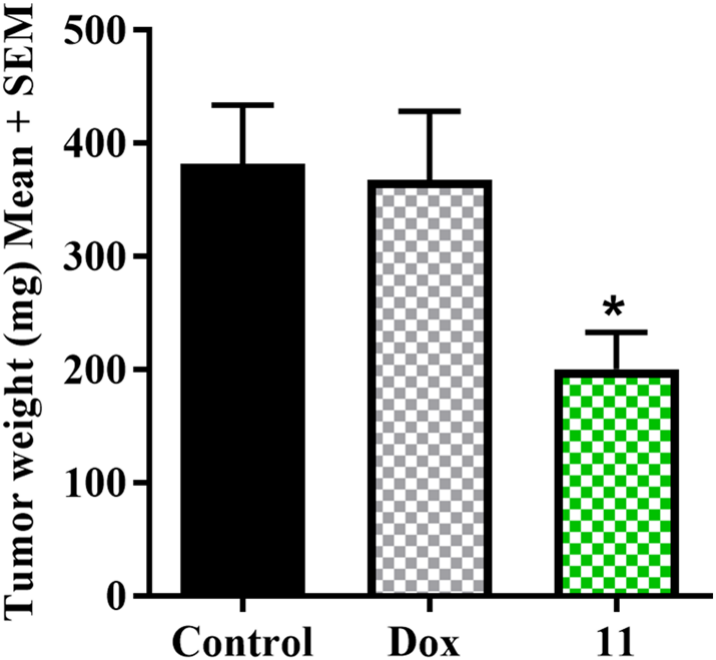
Anticancer efficacy of lead compound **11** and doxorubicin (Dox) in 4T1 tumor syngraft model. Two-factor anova statistical analysis was utilized to compare tumor growth between treated groups and the vehicle control (*P < 0.05).

To further explore the potential of its use as an anticancer agent, we evaluated the tumor growth inhibition effects of compound **11** in a highly aggressive stage IV breast cancer 4T1 mouse tumor model that recapitulates human cancers and can be studied in a non-genetically modified syngraft setting. 5 × 10^4^ 4T1 cells (suspended in 1:1 mixture of PBS-matrigel) were inoculated subcutaneously into the right flank of female BALB/c mice, and treatment was initiated the following day. Mice were then randomly grouped based on body weight. Group 1 was treated with **11** (25 mg/kg, i.p., once daily), group 2 was administered with clinically used breast cancer drug doxorubicin (0.5 mg/kg, i.p., three times a week) and group 3 was administered vehicle (10% DMSO, 10% PEG, 40% HS-15 (18.8%w/v in H_2_O), and 40% H_2_O). The treatment was continued for 17 days and at the end of the study, tumors were resected and weighed where it was found that compound **11** exhibited significant tumor growth reduction (48%) compared to the control group and doxorubicin (Fig. 8). In a parallel fashion, treatment tolerability was observed similar to systemic toxicity study where candidate compounds were again well-tolerated as evidenced by body weight gains, activity, and grooming patterns. Further, it is notable that during optimization of the presented 4T1 tumor model, we had administered doxorubicin at higher concentrations (2–4 mg/kg) which resulted in intolerable levels of tumor ulcerations; disqualifying higher dosing strategies to be performed in the present 4T1 syngraft study. In fact, extensive internalization of tumors along with substantial ulcerations in the study limited tumor volume measurements and for that reason, end-point tumor masses were used as the reporter of treatment efficacy (Fig. 8).

## Discussion

The tumor microenvironment is highly hetereogenous, comprised of numerous tissue types including epithelial cancer cells, stromal cells, immune cells and endothelial cells. Endothelial cells, in particular, play a vital role in angiogenesis for the supply of nutrients and oxygen throughout the rapidly dividing tumor^25^. Hence, numerous attempts have been made to develop angiogenesis inhibitors—with effective agents targeting various endothelial growth factors and receptors^26^. In this regard, we have designed and synthesized a novel class of *N*-phenylindazole containing diarylureas as potential antiangiogenic anticancer agents (Figs. 2, 3). Due to the structural similarity of our candidate compounds with other diphenylurea containing clinical angiogenesis inhibitors such as sorafenib and regorafenib, we explored the potential of candidate compounds **8i** and **11** to inhibit the tube forming ability of endothelial cells in a widely employed in vitro model of HUVEC cells. The tube formation assay is a widely used model to study angiogenesis^24^. This assay evaluates the capability of endothelial cells to divide and migrate in response to angiogenic signals, and test compounds can be assessed for their inhibitory effects on these processes. Specifically, the in vitro HUVEC model employs angiogenic vascular endothelial growth factor (VEGF) to induce and differentiate endothelial cells to form tube-like structures on a supporting matrix. Hence, alterations in the tube forming ability of these cells may indicate dysfunction in VEGF stimulatory pathways. Here, we illustrated that the lead candidate compounds reduced the tube length of HUVEC cells at non-toxic concentrations and hence, may be working through inhibition of angiogenesis and related processes (Fig. 5). Nonetheless, additional in vitro and in vivo experiments are needed to fully evaluate the molecular mechanisms of the candidate compounds. Furthermore, the tube length effects of candidate compounds in HUVEC cells may be independent of the mechanism of cancer cell death in vitro (Table 1) as serum and nutrient rich mono-cultures are not dependent on endothelial cell growth or VEGF stimulated processes for proliferation. Additionally, mechanism of compound induced cell death (apoptosis or other means) in in vivo systems where 3D-heterogenous cell types interact to promote tumor growth is also likely to be different than either in vitro HUVEC or cancer cell lines. Hence, it is likely that the candidate compounds are acting via pleiotropic mechanisms of action to elicit their anticancer properties in vitro and in vivo. In fact, diarylurea containing antiangiogenic agents have been found to exhibit potency across numerous growth stimulatory kinases^18–23^. In particular, numerous mechanisms of cell death have been reported for both regorafenib and sorafenib ranging from apoptotsis, necrosis, ferroptosis, autophagy, and others; involving numerous reported downstream signaling pathways^27–34^. Specifically, it has been reported that the diphenyl urea template targets numerous growth stimulatory pathways including RAS-RAF/MEK-ERK along with the PI3K-mTOR axis to provide its anticancer activity^35^. Additionally, these compounds have been shown to target platelet derived growth factors and receptors and epidermal growth factors and receptors; expanding on the hypothesized VEGF/R mechanisms^35^. Finally, the diphenyl urea template has been utilized in developing HDAC inhibitors, Aurora kinase inhibitors, and also as DNA-directing alkylating agents^35^. Based on these reports, we believe that our candidate compounds may also exhibit pleotropic mechanisms of action in inhibiting cell viability in vitro and tumor growth inhibition in vivo with a combination of cytotoxic and static effects depending on the tissue type. Nonetheless, in the present study we have designed, synthesized, and evaluated a new-class of anticancer agents that exhibit potent effects on cancer cell viability in vitro and have illustrated significant tumor growth inhibition activity in two aggressive solid tumor models of colorectal and breast cancer.

## Conclusion

In conclusion, we have synthesized novel diphenyl indazole containing diarylurea compounds as potential anticancer agents for difficult to treat solid tumors. The synthesized compounds were evaluated against various cancer cell lines including murine metastatic breast cancer 4T1, murine glioma GL261-luc2, human triple negative breast cancer MDA-MB-231, human pancreatic cancer MIAPaCa-2, and human colorectal adenocarcinoma WiDr. Several of the tested candidates inhibited the cell viability in low micromolar concentrations. Based on these studies, three candidate compounds **8i**, **10**, and **11** were selected and further evaluated in vivo in colorectal adenocarcinoma WiDr xenograft model. These studies indicated that candidate **11** was superior in arresting tumor growth compared to **8i** and **10**. The lead candidate **11** was also evaluated for its in vivo efficacy in aggressive stage IV breast cancer 4T1 syngraft model, where it significantly reduced the tumor growth. Since diarylurea based drug candidates are known to exhibit antiangiogenic properties, we further studied candidate compounds **8i** and **11** for their preliminary in vitro mechanism of action. These studies indicated that candidate compounds reduced tube length in endothelial HUVEC cells at sub-toxic concentrations. Further structure activity relationship studies are required to synthesize and identify a more potent lead compound that can completely suppress the tumor growth, wherein detailed mechanism of action studies can be carried out. However, the studies described in the manuscript provide a novel indazole diarylurea structural template which can be further developed for anticancer applications.

## Materials and methods

### Cell lines and culture conditions

Human Umbilical Vein Endothelial Cells (HUVEC) were procured from ATCC and grown in complete Vascular Cell Basal Medium supplemented with Endothelial Cell Growth Kit-VEGF (ATCC). 4T1 cells (ATCC) were grown in RPMI-1640 (corning) supplemented with 10% fetal bovine serum (FBS) and penicillin–streptomycin (50 U/ml, 50 µg/ml). GL261-luc2 cells (Perkin Elmer) were grown in DMEM medium (corning) supplemented with FBS (10%), and penicillin–streptomycin (50 U/ml, 50 µg/ml) with Geneticin (50 µg/ml) as selection marker. MCF7 cells (ATCC) were grown in α-MEM with FBS (5%), non-essential amino acids (0.1 mM), insulin (10 µg/mL), sodium pyruvate (1 mM), epidermal growth factor (100 ng/mL), hydrocortisone (10 µg/mL), HEPES (10 mM), and penicillin–streptomycin (50U/ml, 50 µg/ml). MDA-MB-231 cells (ATCC) were grown in DMEM medium supplemented with FBS (10%) and penicillin–streptomycin (50 U/ml, 50 µg/ml). MIAPaCa-2 cells (ATCC) were grown in DMEM medium supplemented with FBS (10%), horse serum (2.5%) and penicillin–streptomycin (50 U/ml, 50 µg/ml). WiDr cells (ATCC) were cultured in MEM media supplemented with FBS (10%) and penicillin–streptomycin (50 U/ml, 50 µg/ml).

### MTT based cell viability assay

Cellular viability in the presence of test compounds in 4T1, GL261-luc2, MDA-MB-231, MIAPaCa-2, and WiDr cancer cells was determined using MTT (3-(4, 5-dimethylthiazolyl-2)-2, 5-diphenyltetrazolium bromide) assay as previously described by us^36^. Dose response curves in Fig. 4 were generated using GraphPad Prism 7.04, and were analyzed by means of non-linear regression using a non-constrained/non-normalized fit model and were parameterized by the log(concentration inhibitor) against response, where the response represented the percent cell survival when compared to untreated cells. IC_50_ values were extrapolated from the Prism 7.04 model fit, and represent the concentration of compound resulting in 50% cell survival between %survival-maximum and %survival-minimum of the resulting logarithmic fit curve. All compounds were tested at the same concentrations (50–0.39 µM), where resulting IC_50_ values were compared.

### In vitro HUVEC tube-forming assay

In vitro tube formation assay measures the ability of HUVEC cells to form capillary-like structures (called tubes) once plated on extracellular matrix support. The endothelial cells attach and generate mechanical forces on the surrounding extracellular support matrix to create tracks or guidance pathways that facilitate cellular migration. The resulting cords of cells will eventually form hollow lumens. The experiments were performed exactly described by manufacturer's protocol. Briefly, first one layer of extracellular matrix was made by adding 50 μL/well of matrigel in 96 well plates. The plate was incubated for 45 min at 37 °C. The HUVEC cells (2700 cells/ well) were seeded on top of matrigel and incubated for 4 h to form tubes upon stimulation with VEGF supplement. After 4 h the plate was investigated for tube formation. Once tube was formed, cells were treated with 1 µM and 5 µM of compound **8i** and **11** for 12 h. After incubation, cells were stained with Calcein AM, and image was captured. The tube length was measured using Image J software. Briefly, the Image J software program was opened, and then the file to measure the tube length was selected. The tube length was measured using “draw line” icon. The line was drawn on top of tube, next “analyze” icon was selected, and the length of all drawn lines was measured and summed. The data were expressed as the means ± SD of the tube length. At least 10 images from each well were captured and analyzed.

### Systemic toxicity in CD-1 mice

CD-1 mice (Charles River) were randomly assigned into groups (n = 6 mice per group) and mice were administered with compounds **8i**, **10** and **11** as indicated in the results section. Body weights were recorded every day initially and followed by 2–5 days for the duration of the study. At the end of the study, mice were euthanized.

### Tumor growth inhibition studies

5 × 10^6^ WiDr cells were suspended in 100µL (1:1 PBS: Matrigel) (Corning, cat. no. 356237) and were injected subcutaneously into the right flank of 4-week-old female athymic nude mice (Crl:NU(NCr)-Foxn1nu, Charles River). Tumor volume was calculated by measuring the diameter of the long side (a) and the diameter perpendicular (b) where V = ab^2^/2. Tumors were allowed to grow until a mean of ~ 150 mm^3^ was achieved. Mice were then assigned into groups (n = 6 per group). Tumors were measured every two or three days. Groups were assigned as follows: compound **8i** (25 mg/kg, ip, qd), compound **10** (25 mg/kg, ip, qd), compound **11** (25 mg/kg, ip, qd), oxaliplatin (10 mg/kg, ip, qd), and vehicle (10% DMSO, 10% PEG, 40% HS-15 (18.8% w/v in H_2_O), and 40% H_2_O. Intravenous oxaliplatin was not employed to avoid possible tail necrosis in line with our IACUC protocol and as also performed in several literature reports^37–39^. Additionally, since our lead candidate compounds were administered intraperitoneally, we envisioned to administer oxaliplatin also in a similar fashion. The study ended after 14 days or when tumors surpassed acceptable IACUC standards. At the completion of the study tumors were resected and weighed.

5 × 10^4^ 4T1 cells were suspended in 100 µL (1:1 PBS: Matrigel) and were injected subcutaneously into the right flank of 4-week-old female BALB/c (BALB/cAnNCrl, Charles River). The following day, mice were randomly assigned into various groups (n = 8 mice per group) and the treatment was initiated. The compounds were administrated once daily, intraperitoneally. Group 1 was treated with compound 11 (25 mg/Kg), group 2 was given doxorubicin (0.5 mg/Kg, three times a week)^40^, and group 3 was designated as control group and injected with vehicle. The compounds were solubilized in a mixture of 10% mL DMSO, 10% PEG, 40% HS15 (18.8% w/v in sterile water) and 40% sterile water. At the completion of the study tumors were resected and weighed.

### Statistical analysis

Statistical analysis was conducted using GraphPad Prism version 7.04. 2-factor ANOVA was used for in vivo studies. Kruskal–Wallis one-way ANOVA statistical tests were utilized in HUVEC tube-forming assays. In all cases, a *P*-value of < 0.05 was considered significant, and replicates are mentioned in the text and or legends accompanying the figures.

### Ethical approval

The systemic toxicity and anticancer efficacy studies were approved and in compliance with the University of Minnesota’s Institutional Animal Care and Use Committee [Systemic toxicity 1611-34326A (Fig. 6); WiDr xenograft 1612-34444A (Fig. 7), and 4T1 syngraft 1706-34857A (Fig. 8)]. All studies were performed in accordance with the relevant guidelines and regulations, and all protocols were approved by the University of Minnesota.

## Supplementary information

Supplementary Information.
