# Supplementary material for: Synthesis, in vitro, and in vivo evaluation of novel N-phenylindazolyl diarylureas as potential anti-cancer agents
Source: Sci Rep. 2020 Oct 21;10:17969. doi: 10.1038/s41598-020-74572-1 (PMC7578069; doi:10.1038/s41598-020-74572-1)

Supplemental Information

Synthesis, *in vitro*, and *in vivo* evaluation of novel N-phenylindazolyl diarylureas as potential anti-cancer agents

Lucas N. Solano[a], Grady L. Nelson[a], Conor T. Ronayne[a], Shirisha Jonnalagadda[a], Sravan K. Jonnalagadda[a], Kaija Kottke[b], Robert Chitren[c], Joseph L. Johnson[b,e], Manoj K. Pandey[d], Subash C. Jonnalagadda[c], and Venkatram R. Mereddy*[a,b,e]

[a]Integrated Biosciences Graduate Program, University of Minnesota, Duluth, MN 55812
[b]Department of Chemistry and Biochemistry, University of Minnesota Duluth, Duluth, MN 55812
[c]Department of Biomedical Sciences, Cooper Medical School of Rowan University, Camden, NJ 08103
[d]Department of Chemistry and Biochemistry, Rowan University, Glassboro, NJ 08028
[e]Department of Pharmacy Practice & Pharmaceutical Sciences, University of Minnesota, Duluth, MN 55812

*Correspondence to [vmereddy@d.umn.edu]

Preparation of methyl 1-(4-nitrophenyl)-1H-indazole-3-carboxylate (3)

Synthesis of the precursor **3** was conducted by taking commercially available methyl 1H-indazole-3-carboxylate (10 mmol) and dissolving it in 50mL of DMSO. 1-fluoro-4-nitrobenzene (11 mmol) and potassium carbonate (30 mmol) were added to the solution, and the mixture was heated to 120°C for 4hr. After cooling to room temperature, the solution was poured over ice and the resulting pale yellow solid **3** was filtered, dried, and then stirred in hexanes followed by filtration a second time to afford the pure product in 84% yield.

^{1}H NMR (500 MHz, DMSO-d_6) δ 8.45 (d, *J* = 9 Hz, 2H), 8.22 (d, *J* = 8 Hz, 1H), 8.14 (d, *J* = 9 Hz, 2H), 8.05 (d, *J* = 9 Hz, 1H), 7.66 (t, *J* = 8 Hz, 1H), 7.51 (t, *J* = 8 Hz, 1H), 3.99 (s, 3H)

^{13}C NMR (126 MHz, DMSO-d_6) δ 162.3, 146.2, 144.2, 139.9, 138.2, 129.3, 125.8, 125.1, 124.6, 123.5, 122.5, 112.2, 52.7

Preparation of methyl 1-(4-aminophenyl)-1H-indazole-3-carboxylate (4):

Synthesis of **4** was conducted by taking **3** (10 mmol) and dissolving it in THF 50mL. Pd/C (0.5 g, 10% Pd) and ammonium formate (30 mmol) were added and the mixture was refluxed for 4hr. After completion of the reaction the mixture was filtered through a pad of silica gel and the solution was evaporated under reduced pressure to afford the pure product in 89% yield.

^{1}H NMR (500 MHz, DMSO-d_6) δ 8.16 (d, *J* = 8 Hz, 1H), 7.67 (d, *J* = 9 Hz, 1H), 7.5 (t, *J*=7 Hz, 1H), 7.36 - 7.40 (m, 3H), 6.68 - 6.82 (m, 2H), 5.53 (s, 2H), 3.95 (s, 3H)

^{13}C NMR (126 MHz, DMSO-d_6) δ 162.9, 149.6, 140.3, 135.0, 127.9, 127.6, 125.4, 124.0, 123.8, 121.8, 114.4, 111.8, 52.2

Preparation of ureido benzoic acids (7a-k)

4-Aminobenzoic acid (10 mmol) and respective isocyante (10 mmol) were dissolved separately in THF (25mL). The two solutions were then mixed together and a precipitate formed over 4 hours. The precipitate was filtered through Buchner funnel and washed with THF to afford the pure products **7a-k** (69%-91% yield).

Preparation of methyl 1-(4-(4-(3-phenylureido)benzamido)phenyl)-1H-indazole-3-carboxylates (8a-k)

To a solution of respective ureido benzoic acids (**7a-k**, 10 mmol) in DMF (50 mL) was added triethylamine (30 mmol), EDC (11 mmol), and HOBt (11 mmol). Reaction mixture was stirred for 10 min before the addition of methyl 1-(4-aminophenyl)-1H-indazole-3-carboxylate (**4**) (10.5 mmol). Reaction was warmed to 60 °C and stirred overnight. After completion of the reaction, mixture was poured over ice, and the resulting crude product was filtered and washed with repeatedly with water to remove DMF and excess EDC. The resulting crude product was recrystallized to obtain pure products **8a-k**.

Methyl 1-(4-(4-(3-phenylureido)benzamido)phenyl)-1H-indazole-3-carboxylate (8a)

Yield: 82%

^{1}H NMR (500 MHz, DMSO-d_6) δ 10.39 (br. s., 1H), 9.06 (br. s., 1H), 8.81 (br. s., 1H), 8.23 (d, *J* = 8 Hz, 1H), 8.08 - 8.11 (m, 2H), 7.99 - 8.02 (m, 2H), 7.90 (d, *J* = 8 Hz, 1H), 7.82 (br. s., 2H), 7.61 - 7.67 (m, 3H), 7.50 (d, *J* = 6 Hz, 3H), 7.33 (d, *J* = 7 Hz, 2H), 7.02 (d, *J* = 6 Hz, 1H), 3.99 (s, 3H)

^{13}C NMR (126 MHz, DMSO-d_6) δ 165.6, 162.8, 152.8, 143.6, 140.1, 139.9, 139.7, 136.2, 134.3, 129.4, 129.3, 128.5, 127.9, 124.5, 124.2, 124.1, 122.6, 122.1, 121.5, 118.9, 117.6, 111.9, 52.4

HRMS (ESI) m/z: calc'd for $C_{29}H_{23}N_5O_4$ $[M+H]^+$: 506.1823, found 506.1556

Methyl 1-(4-(4-(3-(2-chlorophenyl))benzamido)phenyl)-1H-indazole-3-carboxylate (8b):

Yield: 87%

^{1}H NMR (500 MHz, DMSO-d_6) δ 10.41 (s, 1H), 9.77 (s, 1H), 8.44 (s, 1H), 8.19 (d, *J* = 8 Hz, 1H), 8.22 (d, *J* = 8 Hz, 1H), 8.09 (d, *J* = 9 Hz, 2H), 8.02 (d, *J* = 9 Hz, 2H), 7.88 (d, *J* = 9 Hz, 1H), 7.80 (d, *J* = 9 Hz, 2H), 7.56 - 7.72 (m, 3H), 7.44 - 7.53 (m, 2H), 7.33 (t, *J* = 8 Hz, 1H), 7.07 (t, *J* = 8 Hz, 1H), 3.99 (s, 3H)

^{13}C NMR (126 MHz, DMSO-d_6) δ 165.6, 162.8, 152.4, 143.3, 140.1, 139.7, 136.2, 136.1, 134.3, 129.7, 129.5, 128.4, 128.3, 128.1, 124.4, 124.2, 124.1, 124.1, 122.7, 122.1, 122.0, 121.5, 117.7, 111.9, 52.4

HRMS (ESI) m/z: calc'd for $C_{29}H_{22}ClN_5O_4$ $[M+H]^+$: 540.1433, found 540.1127

Methyl 1-(4-(4-(3-(4-tolyl))benzamido)phenyl)-1H-indazole-3-carboxylate (8c):

Yield: 84%

^{1}H NMR (500 MHz, DMSO-d_6) δ 10.37 (s, 1H), 9.00 (s, 1H), 8.69 (s, 1H), 8.22 (d, *J* = 8 Hz, 1H), 8.08 (d, *J* = 9 Hz, 2H), 7.98 (d, *J* = 9 Hz, 2H), 7.88 (d, *J* = 8 Hz, 1H), 7.80 (d, *J* = 9 Hz, 2H), 7.55 - 7.67 (m, 3H), 7.43 - 7.50 (m, 1H), 7.37 (d, *J* = 8 Hz, 2H), 7.11 (d, *J* = 8 Hz, 2H), 3.99 (s, 3H), 2.26 (s, 3H)

^{13}C NMR (126 MHz, DMSO-d_6) δ 165.6, 162.8, 152.8, 143.7, 140.1, 139.7, 137.3, 136.2, 134.3, 131.5, 129.7, 129.4, 128.5, 127.8, 124.5, 124.2, 124.1, 122.1, 121.5, 119.0, 117.6, 111.9, 52.4, 20.8

HRMS (ESI) m/z: calc'd for $C_{30}H_{25}N_5O_4$ $[M+H]^+$: 520.1979, found 520.1707

Methyl 1-(4-(4-(3-(4-(trifluromethyl))benzamido)phenyl)-1H-indazole-3-carboxylate (8d):
Yield: 84%

^{1}H NMR (500 MHz, DMSO-d_6) δ 10.40 (s, 1H), 9.22 (s, 1H), 9.18 (s, 1H), 8.22 (d, *J* = 8 Hz, 1H), 8.08 (d, *J* = 9 Hz, 2H), 8.00 (d, *J* = 9 Hz, 2H), 7.88 (d, *J* = 9 Hz, 1H), 7.80 (d, *J* = 9 Hz, 2H), 7.62 - 7.72 (m, 6H), 7.59 (t, *J* = 7 Hz, 1H), 7.47 (t, *J* = 7 Hz, 1H), 3.99 (s, 3H)

^{13}C NMR (126 MHz, DMSO-d_6) δ 165.6, 162.8, 152.6, 143.7, 143.2, 140.1, 139.7, 136.2, 134.3, 129.4, 128.5, 128.3, 126.6,126.1,126.1, 124.3 (q), 122.6 (q), 122.1, 121.5, 118.5, 117.9, 111.9, 52.4

HRMS (ESI) m/z: calc'd for $C_{30}H_{22}F_3N_5O_4$ $[M+H]^+$: 574.1697, found 574.1376

Methyl 1-(4-(4-(3-(4-methoxyphenyl)benzamido)phenyl)-1H-indazole-3-carboxylate (8e):
Yield: 82%

^{1}H NMR (500 MHz, DMSO-d_6) δ 10.37 (s, 1H), 8.97 (s, 1H), 8.61 (s, 1H), 8.22 (d, *J* = 8 Hz, 1H), 8.08 (d, *J* = 9 Hz, 2H), 7.98 (d, *J* = 8 Hz, 2H), 7.88 (d, *J* = 9 Hz, 1H), 7.80 (d, *J* = 9 Hz, 2H), 7.55 - 7.66 (m, 3H), 7.46 (t, *J* = 8 Hz, 1H), 7.35 - 7.42 (m, 2H), 6.90 (d, J=8 Hz, 2H), 3.99 (s, 3H), 3.73 (s, 3H)

^{13}C NMR (126 MHz, DMSO-d_6) δ 165.7, 162.8, 155.2, 152.9, 143.8, 140.1, 139.7, 136.2, 134.3, 132.8, 129.4, 128.4, 127.7, 124.4, 124.2, 124.1, 122.1, 121.5, 120.7, 117.5, 114.5, 111.9, 55.7, 52.4

HRMS (ESI) m/z: calc'd for $C_{30}H_{25}N_5O_5$ $[M+H]^+$: 536.1928 , found 536.1628

Methyl 1-(4-(4-(3-(4-fluorophenyl)benzamido)phenyl)-1H-indazole-3-carboxylate (8f):
Yield: 82%

^{1}H NMR (500 MHz, DMSO-d_6) δ 10.38 (s, 1H), 9.05 (s, 1H), 8.83 (s, 1H), 8.22 (d, *J* = 8 Hz, 1H), 8.07 (d, *J* = 9 Hz, 2H), 7.98 (d, *J* = 9 Hz, 2H), 7.88 (d, *J* = 9 Hz, 1H), 7.80 (d, *J* = 9 Hz, 2H), 7.55 - 7.67 (m, 3H), 7.40 - 7.53 (m, 3H), 7.09 - 7.21 (m, 2H), 3.99 (s, 3H)

^{13}C NMR (126 MHz, DMSO-d_6) δ 165.6, 162.8,158.9, 157.0, 152.9, 143.6, 140.1, 139.7, 136.2, 134.3, 129.4, 128.5, 127.9, 124.5, 124.2, 124.1, 122.1, 121.5, 120.7, 120.7, 117.7, 115.9, 115.7, 111.9, 52.4

HRMS (ESI) m/z: calc'd for $C_{29}H_{22}FN_5O_4$ $[M+H]^+$: 524.1729, found 524.1447

Methyl 1-(4-(4-(3-(4-chlorophenyl))benzamido)phenyl)-1H-indazole-3-carboxylate (8g):
Yield: 86%

^{1}H NMR (500 MHz, DMSO-d$_6$) δ 10.38 (s, 1H), 9.08 (s, 1H), 8.94 (s, 1H), 8.21 (d, *J* = 8 Hz, 1H), 8.08 (d, *J* = 9 Hz, 2H), 7.99 (d, *J* = 8 Hz, 2H), 7.87 (d, *J* = 8 Hz, 1H), 7.79 (d, *J* = 9 Hz, 2H), 7.63 (d, *J* = 9 Hz, 2H), 7.59 (t, *J* = 8 Hz, 1H), 7.49 - 7.55 (m, 2H), 7.42 - 7.48 (m, 1H), 7.35 (d, *J* = 9 Hz, 2H), 3.98 (s, 3H)

^{13}C NMR (126 MHz, DMSO-d$_6$) δ 165.6, 162.8, 152.7, 143.4, 140.1, 139.7, 138.9, 136.2, 134.3, 129.4, 129.1, 128.4, 128.1, 126.1, 124.4, 124.2, 124.1, 122.1, 121.5, 120.4, 117.8, 111.9, 52.4

HRMS (ESI) m/z: calc'd for $C_{29}H_{22}ClN_5O_4$ [M+H]$^+$: 540.1433, found 540.1137

Methyl 1-(4-(4-(3-(2,4-dichlorophenyl))benzamido)phenyl)-1H-indazole-3-carboxylate (8h):
Yield: 78%

^{1}H NMR (500 MHz, DMSO-d$_6$) δ 10.40 (s, 1H), 9.85 (s, 1H), 8.57 (s, 1H), 8.18 - 8.24 (m, 2H), 8.05 - 8.11 (m, 2H), 8.01 (d, *J* = 9 Hz, 2H), 7.88 (d, *J* = 9 Hz, 1H), 7.77 - 7.82 (m, 2H), 7.61 - 7.67 (m, 3H), 7.55 - 7.61 (m, 1H), 7.44 - 7.49 (m, 1H), 7.41 (d, *J* = 9 Hz, 1H), 3.98 (s, 3H)

^{13}C NMR (126 MHz, DMSO-d$_6$) δ 165.6, 162.8, 152.3, 143.1, 140.1, 139.7, 136.2, 135.4, 134.3, 129.5, 129.1, 128.5, 128.4, 128.2, 126.9, 124.5, 124.2, 124.1, 123.4, 122.8, 122.1, 121.5, 117.8, 111.9, 52.4

HRMS (ESI) m/z: calc'd for $C_{29}H_{21}$ $Cl_2N_5O_4$ [M+H]$^+$: 574.1043 , found 574.0724

Methyl 1-(4-(4-(3-(4-chloro-3-(trifluoromethyl)phenyl) benzamido) phenyl)-1H-indazole-3-carboxylate (8i):

Yield: 81%

^{1}H NMR (500 MHz, DMSO-d$_6$) δ 10.40 (s, 1H), 9.28 (s, 1H), 9.22 (s, 1H), 8.18 - 8.24 (m, 1H), 8.14 (s, 1H), 8.08 (d, *J* = 8 Hz, 2H), 8.00 (d, *J* = 8 Hz, 2H), 7.88 (d, *J* = 8 Hz, 1H), 7.80 (d, *J* = 8 Hz, 2H), 7.54 - 7.71 (m, 5H), 7.46 (t, *J* = 8 Hz, 1H), 3.98 (s, 3H)

^{13}C NMR (126 MHz, DMSO-d$_6$) δ 165.6, 162.8, 152.7, 143.1, 140.1, 139.7, 139.5, 136.2, 134.3, 132.5, 129.4, 128.5-127.1(q), 124.4 (q), 124.2, 124.1, 123.7, 123.1, 122.2, 122.1, 121.5, 120.0, 118.1, 117.4, 117.4, 111.9, 52.4

HRMS (ESI) m/z: calc'd for $C_{30}H_{21}ClF_3N_5O_4$ [M+H]$^+$: 608.1307, found 608.1000

Methyl 1-(4-(4-(3-(3,4,5-(trimethoxy)phenyl) benzamido) phenyl)-1H-indazole-3-carboxylate (8j):

Yield: 79%

^{1}H NMR (500 MHz, DMSO-d$_6$) δ 10.37 (s, 1H), 8.99 (s, 1H), 8.74 (s, 1H), 8.22 (d, *J* = 8 Hz, 1H), 8.07 (d, *J* = 9 Hz, 2H), 7.98 (d, *J* = 9 Hz, 2H), 7.88 (d, *J* = 8 Hz, 1H), 7.80 (d, *J* = 9 Hz, 2H), 7.55 - 7.66 (m, 3H), 7.40 - 7.51 (m, 1H), 6.82 (s, 2H), 3.97 (s, 3H), 3.76 (s, 6H), 3.61 (s, 3H)

^{13}C NMR (126 MHz, DMSO-d$_6$) δ 165.6, 162.8, 153.4, 152.7, 143.5, 140.1, 139.7, 136.2, 136.0, 134.3, 133.2, 129.4, 128.4, 127.9, 124.4, 124.2, 124.1, 122.1, 121.5, 117.7, 111.9, 96.7, 60.6, 56.2, 52.4

HRMS (ESI) m/z: calc'd for $C_{32}H_{29}N_5O_7$ $[M+H]^+$: 596.2140 , found 596.1865

Methyl 1-(4-(4-(3-cyclohexylureido))benzamido)phenyl)-1H-indazole-3-carboxylate (8k):

Yield: 83%

^{1}H NMR (500 MHz, DMSO-d$_6$) δ 10.32 (s, 1H), 8.68 (s, 1H), 8.21 (d, *J* = 8 Hz, 1H), 8.07 (d, *J* = 9 Hz, 2H), 7.93 (d, *J* = 9 Hz, 2H), 7.87 (d, *J* = 8 Hz, 1H), 7.78 (d, *J* = 9 Hz, 2H), 7.50 - 7.61 (m, 3H), 7.37 - 7.48 (m, 1H), 6.23 (d, *J* = 8 Hz, 1H), 3.98 (s, 3H), 3.43 - 3.55 (m, 1H), 2.08 (s, 1H), 1.77 - 1.86 (m, 2H), 1.68-1.50 (m, 3H), 1.25 - 1.38 (m, 2H), 1.13 - 1.24 (m, 3H)

^{13}C NMR (126 MHz, DMSO-d$_6$) δ 165.7, 162.8, 154.5, 144.4, 140.1, 139.8, 136.1, 134.2, 129.3, 128.4, 127.0, 124.4, 124.2, 124.1, 122.1, 121.4, 116.9, 111.9, 52.4, 48.2, 33.3, 25.7, 24.8

HRMS (ESI) m/z: calc'd for $C_{29}H_{29}N_5O_4$ $[M+H]^+$: 512.2292, found 512.2034

***1-(4-(4-(3-(4-chloro-3-(trifluoromethyl)phenyl)ureido)benzamido)phenyl)-1H-indazole-3-carboxylic acid* (9)** methyl 1-(4-(4-(3-phenylureido)benzamido)phenyl)-1H-indazole-3-carboxylate (**8i**) (5 mmol) was dissolved in DMSO (30 mL) and 2.5 M NaOH (5mL) was added and the reaction mixture was heated. Upon completion of hydrolysis reaction mixture was poured over ice and acidified with 3 M HCl dropwise. Solid was filtered and recrystallized with acetone to obtain pure **9** in 76% yield.

1-(4-(4-(3-(4-chloro-3-(trifluoromethyl)phenyl)ureido)benzamido)phenyl)-N-(2-(dimethylamino)ethyl)-1H-indazole-3-carboxamide **(10)**

To a solution of *1-(4-(4-(3-(4-chloro-3-(trifluoromethyl)phenyl)ureido)benzamido)phenyl)-1H-indazole-3-carboxylic acid* (**9**) (2 mmol) in DMF (10 mL) was added triethylamine (2.2 mmol), EDC (2.2 mmol), and HOBt (2.2 mmol). Reaction mixture was stirred for 10 min before the addition of N,N′-dimethylethylenediamine (2.5 mmol). Reaction was warmed to 60 °C and stirred overnight. After completion of reaction mixture was poured over ice and filtered. Resulting solid was recrystallized in ethyl acetate to obtain pure product in 81% yield.

^{1}H NMR (500 MHz, DMSO-d_6) δ 10.37 (s, 1H), 9.39 (s, 1H), 9.31 (s, 1H), 8.36 (t, *J* = 6 Hz, 1H), 8.30 (d, *J* = 8 Hz, 1H), 8.13 (s, 1H), 8.05 (d, *J* = 8 Hz, 2H), 7.99 (d, *J* = 8 Hz, 2H), 7.77 - 7.84 (m, 4H), 7.60 - 7.69 (m, 4H), 7.54 (t, *J* = 8 Hz, 1H), 7.37 (t, *J* = 8 Hz, 1H), 3.41 - 3.47 (m, 2H), 2.45 - 2.51 (m, 2H), 2.21 (s, 6H)

^{13}C NMR (126 MHz, DMSO-d6) δ 165.5, 162.1, 152.7, 143.1, 140.1, 139.6, 139.4, 139.3, 134.6, 132.5, 129.3, 128.4, 128.3, 124.0, 123.7(q), 123.6, 123.5, 123.0(q), 122.7, 121.4, 118.0, 117.4, 117.4, 111.4, 58.5, 45.5, 36.9

HRMS (ESI) m/z: calc'd for $C_{33}H_{29}ClF_3N_7O_3$ $[M+H]^+$: 664.2045, found 664.1690

4-(3-(4-chloro-3-(trifluoromethyl)phenyl)ureido)-N-(4-(3-(4-methylpiperazine-1-carbonyl)-1H-indazol-1-yl)phenyl)benzamide **(11)**

To a solution of *1-(4-(4-(3-(4-chloro-3-(trifluoromethyl)phenyl)ureido)benzamido)phenyl)-1H-indazole-3-carboxylic acid* (**9**) (2 mmol) in DMF (10 mL) was added triethylamine (2.2 mmol), EDC (2.2 mmol), and HOBt (2.2 mmol). Reaction mixture was stirred for 10 min before the addition of 1-methylpiperazine (2.5 mmol). Reaction was warmed to 60 °C and stirred overnight. After completion of reaction the mixture was poured over ice and filtered. The resulting crude solid was recrystallized in ethyl acetate to obtain the pure solid in 76% yield.

^{1}H NMR (500 MHz, DMSO-d_6) δ 10.37 (s, 1H), 9.32 (s, 1H), 9.26 (s, 1H), 8.14 (d, *J* = 1.95 Hz, 1H), 8.02 - 8.09 (m, 3H), 8.02 - 8.03 (m, 1H), 7.99 (d, *J* = 8.79 Hz, 2H), 7.85 (d, *J* = 8.30 Hz, 1H), 7.78 (d, *J* = 8.79 Hz, 2H), 7.61 - 7.71 (m, 5H), 7.55 (t, *J* = 7.56 Hz, 1H), 7.32 - 7.40 (m, 1H), 3.97 (br. s., 2H), 3.76 (br. s., 2H), 3.34 (br. s., 1H), 2.33 - 2.46 (m, 4H), 2.22 (s, 3H)

^{13}C NMR (126 MHz, DMSO-d6) δ 165.6, 161.8, 152.7, 143.1, 140.1, 139.6, 139.3 139.1, 134.7, 132.5, 129.3, 128.5, 128.4,127.3(q), 127.1, 123.8, 123.3, 122.4, 122.2, 121.6, 118.1, 117.4, 111.3, 55.7, 54.9, 47.0, 46.1, 42.3, 31.2

HRMS (ESI) m/z: calc'd for $C_{34}H_{29}ClF_3N_7O_3$ $[M+H]^+$: 676.2045, found 676.1673

^{1}H NMR: Compound **8a**

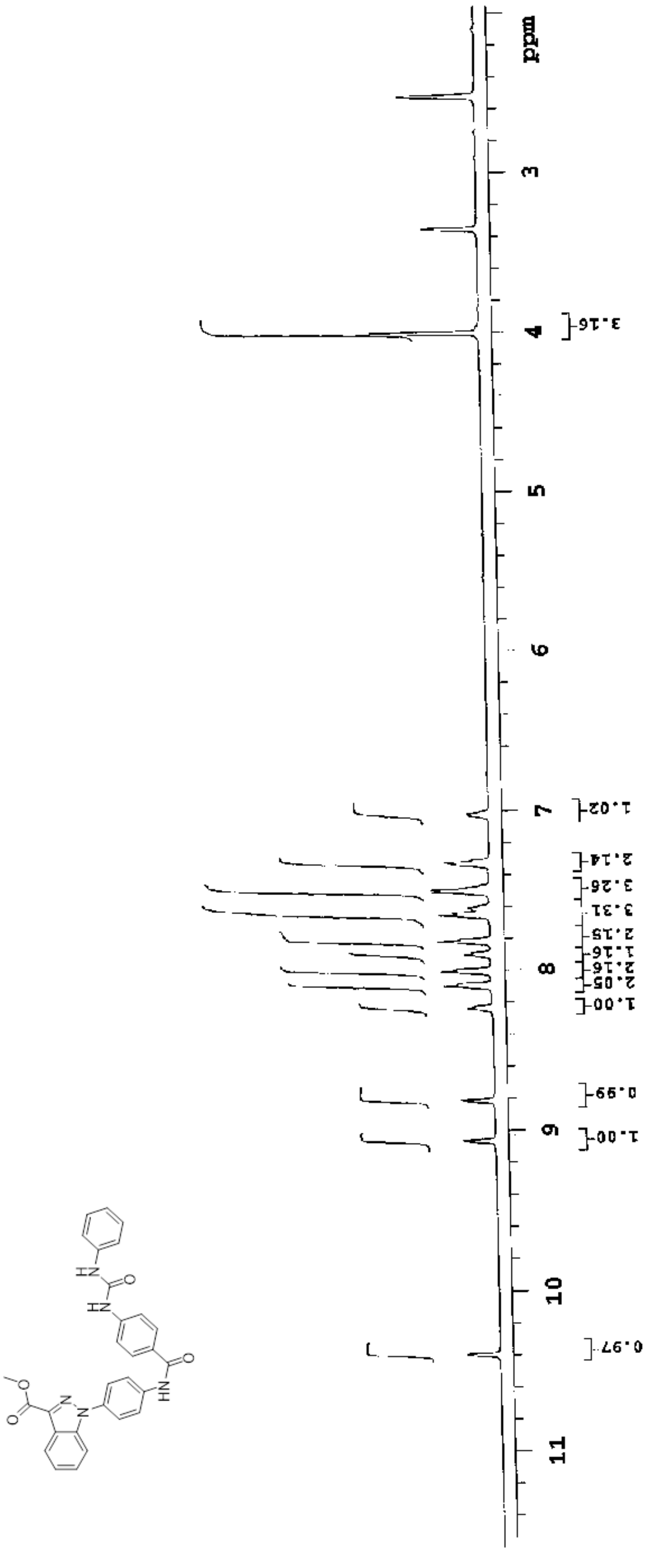

^{13}C NMR: Compound **8a**

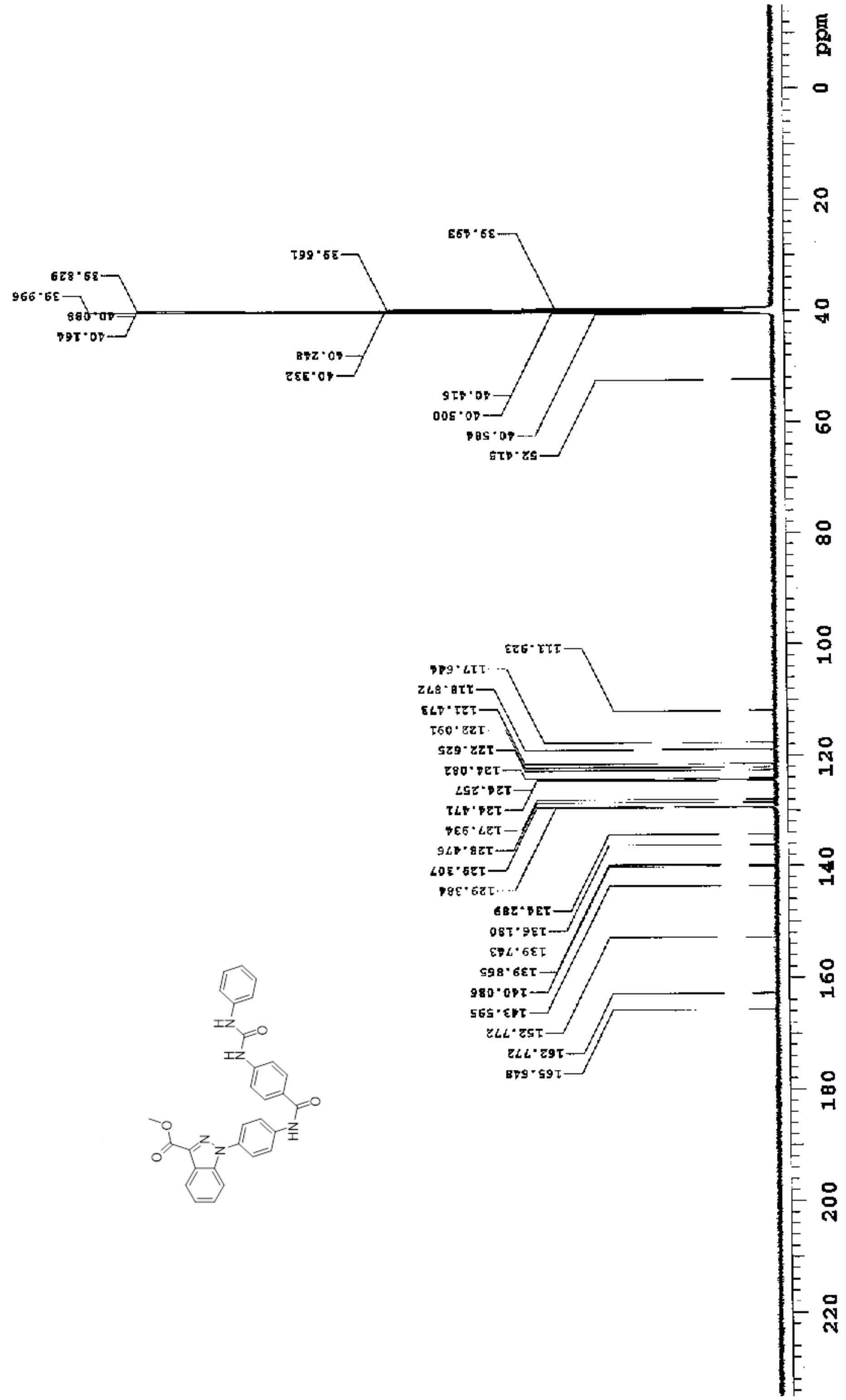

^{1}H NMR: Compound **8b**

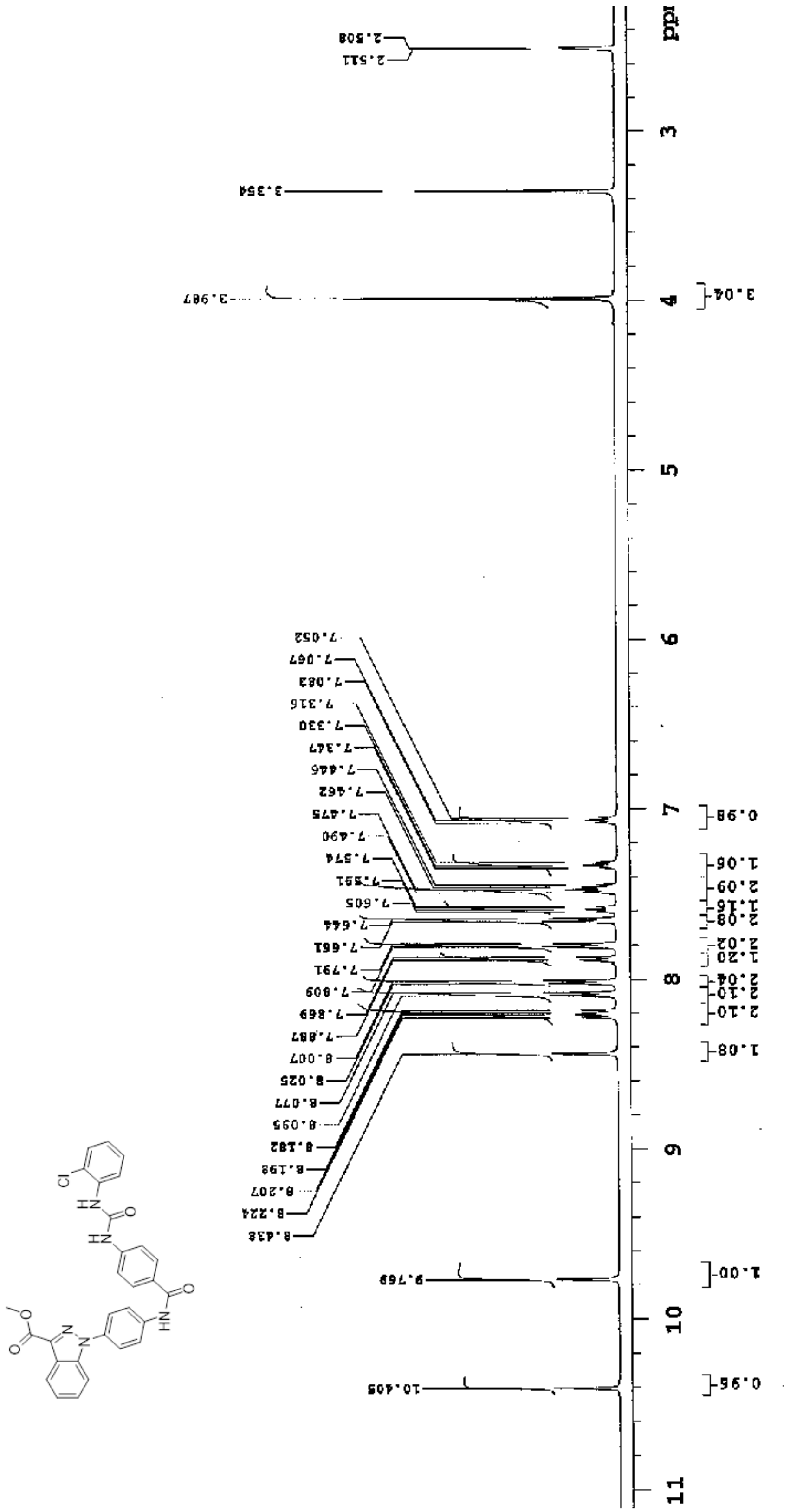

^{13}C NMR: Compound **8b**

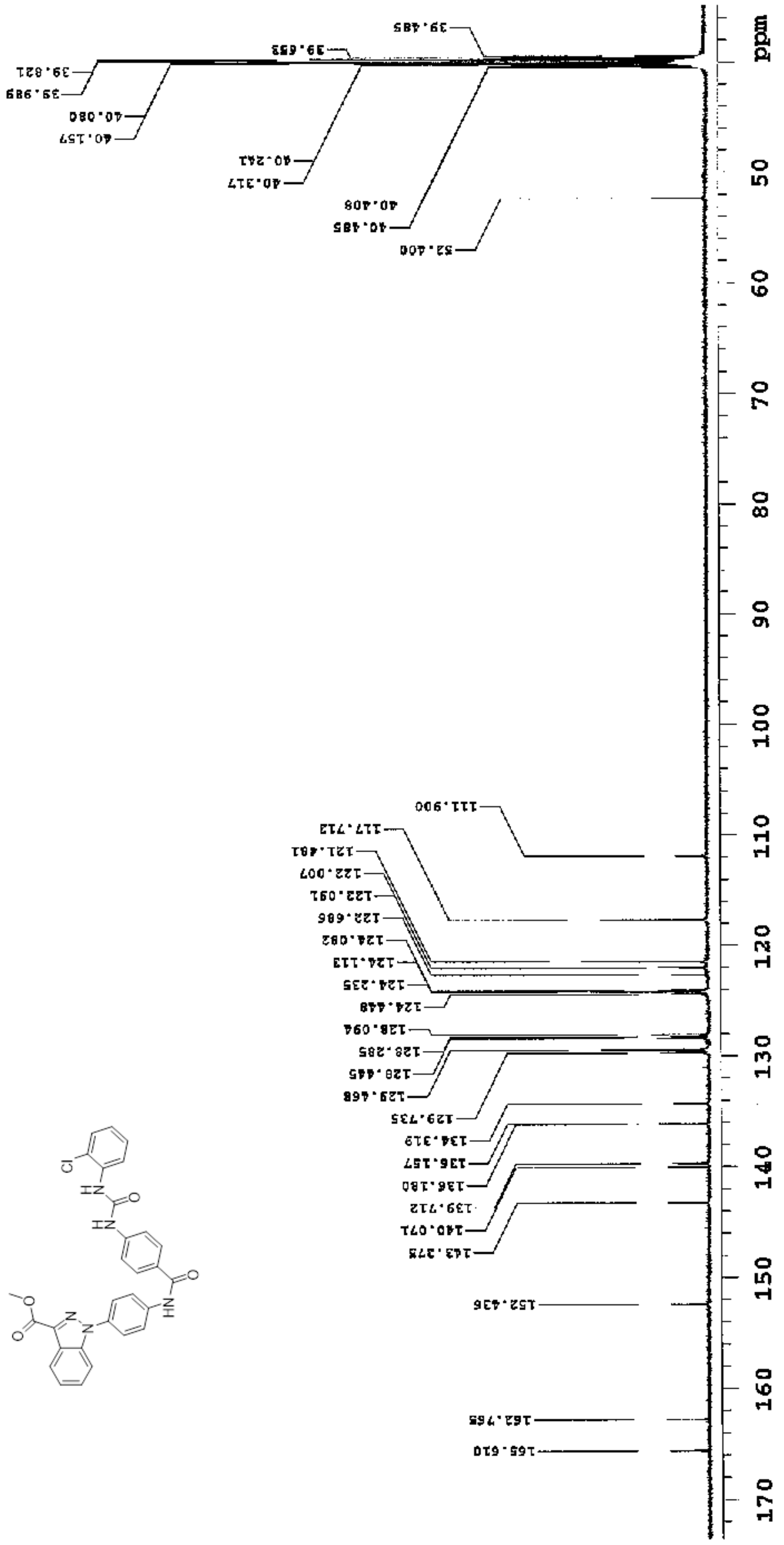

^{1}H NMR: Compound **8c**

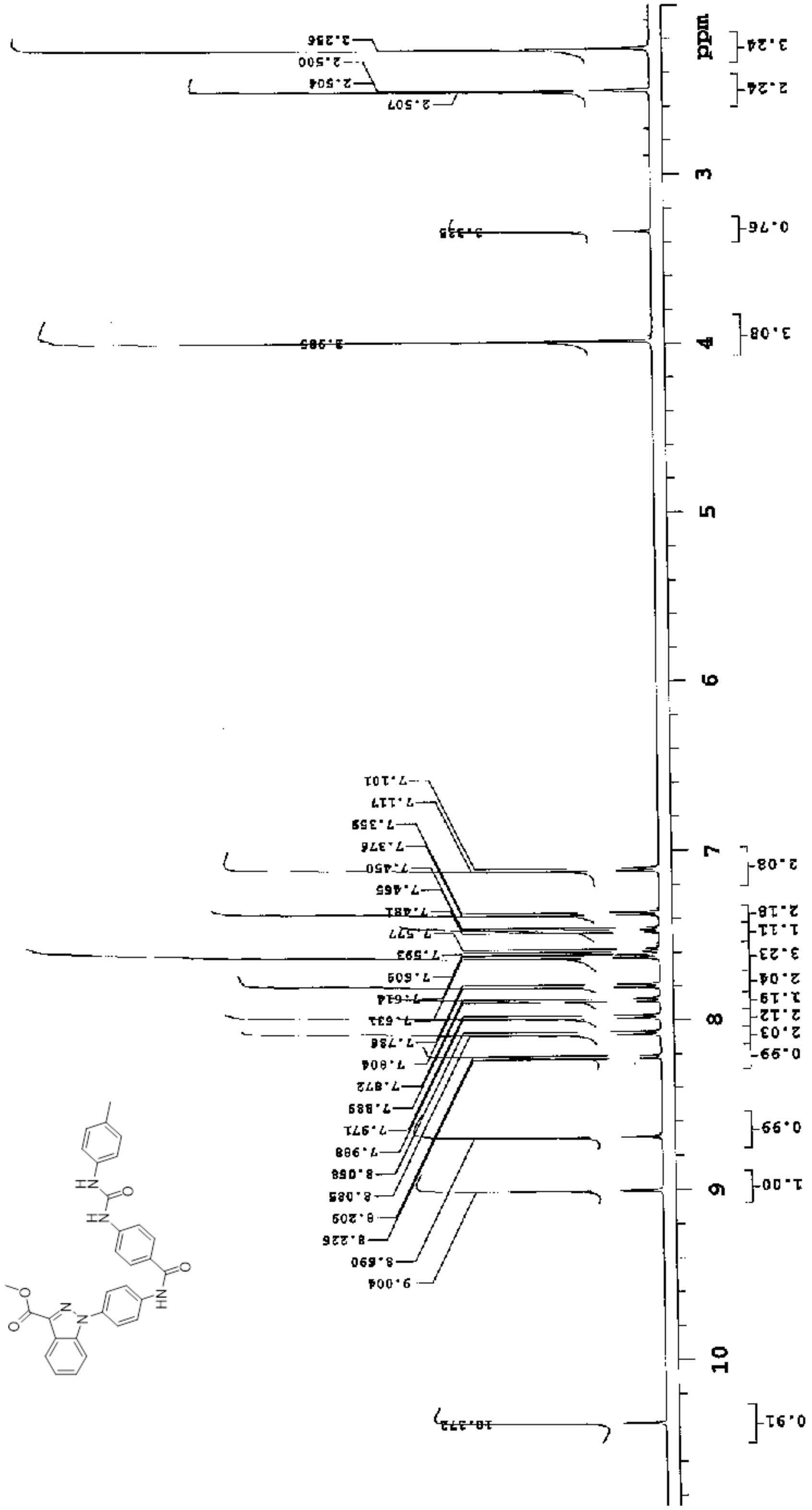

^{13}C NMR: Compound **8c**

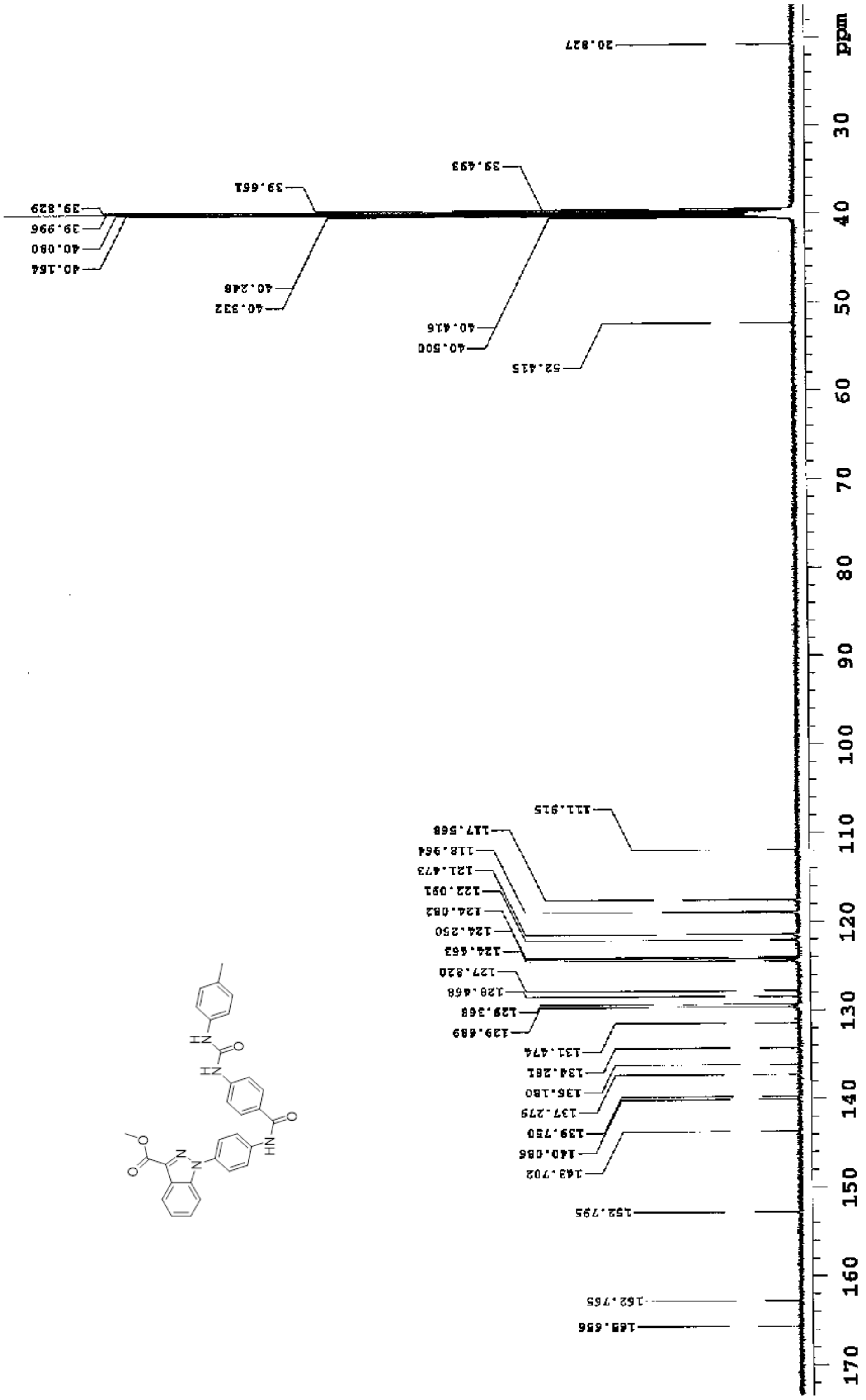

^{1}H NMR: Compound **8d**

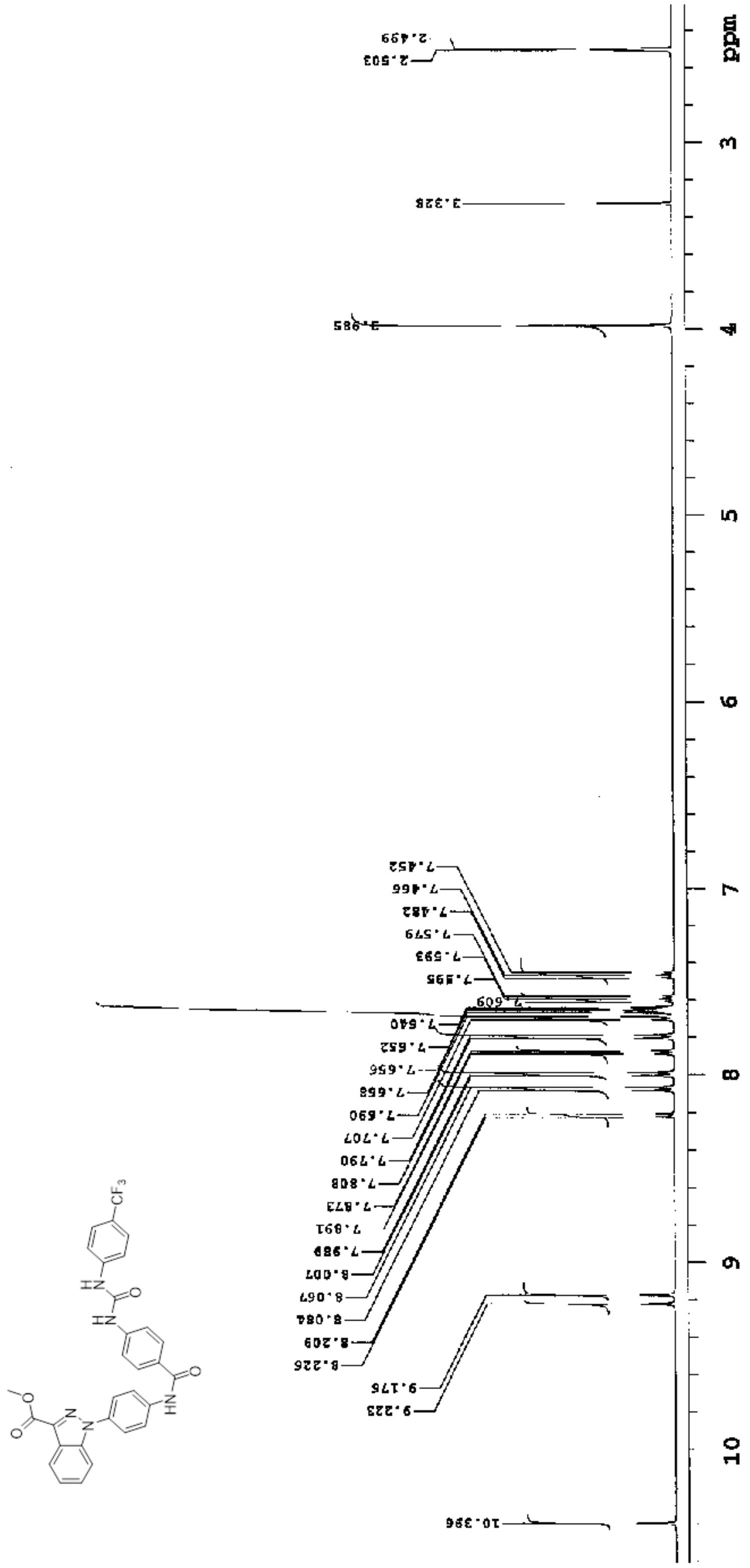

^{13}C NMR: Compound **8d**

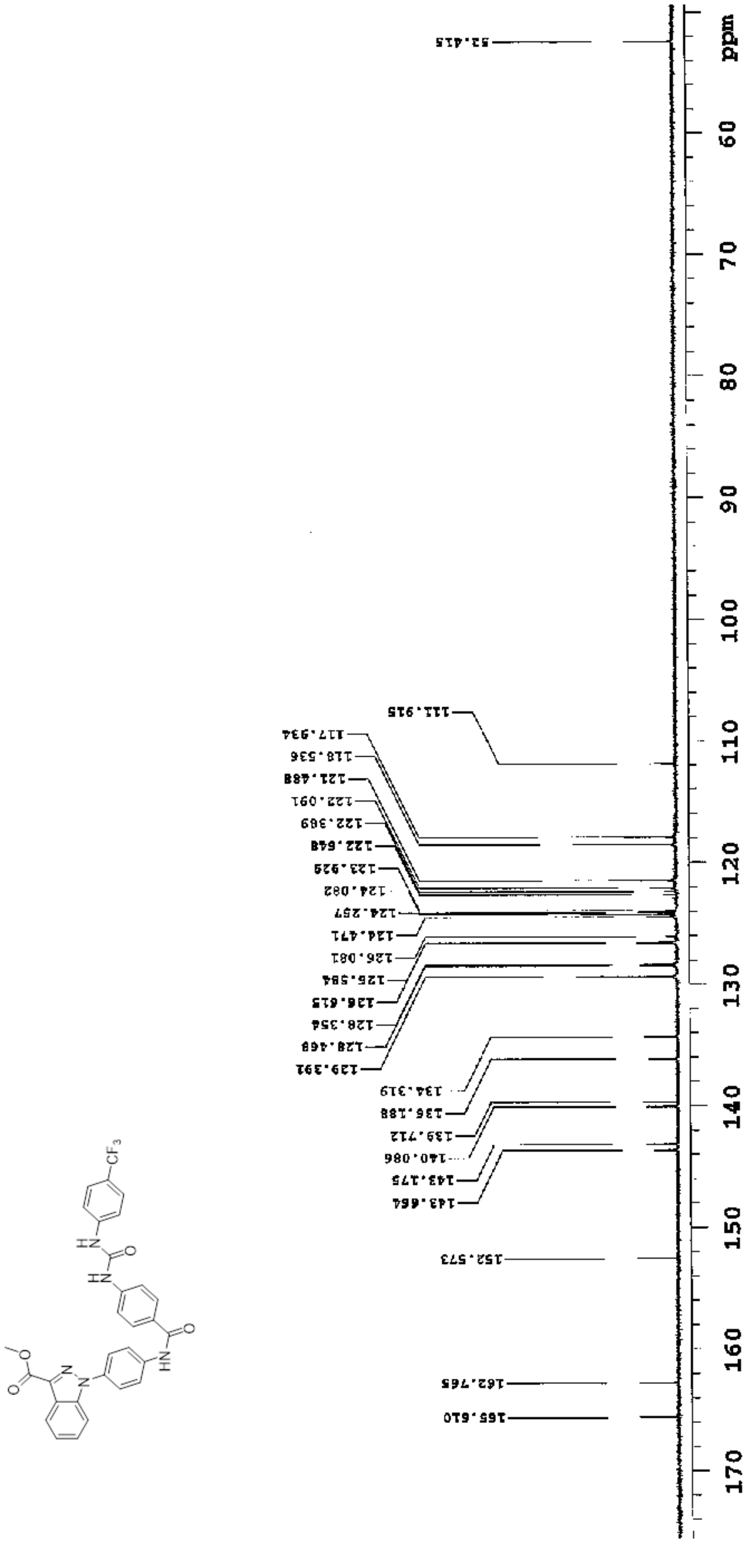

^{1}H NMR: Compound **8e**

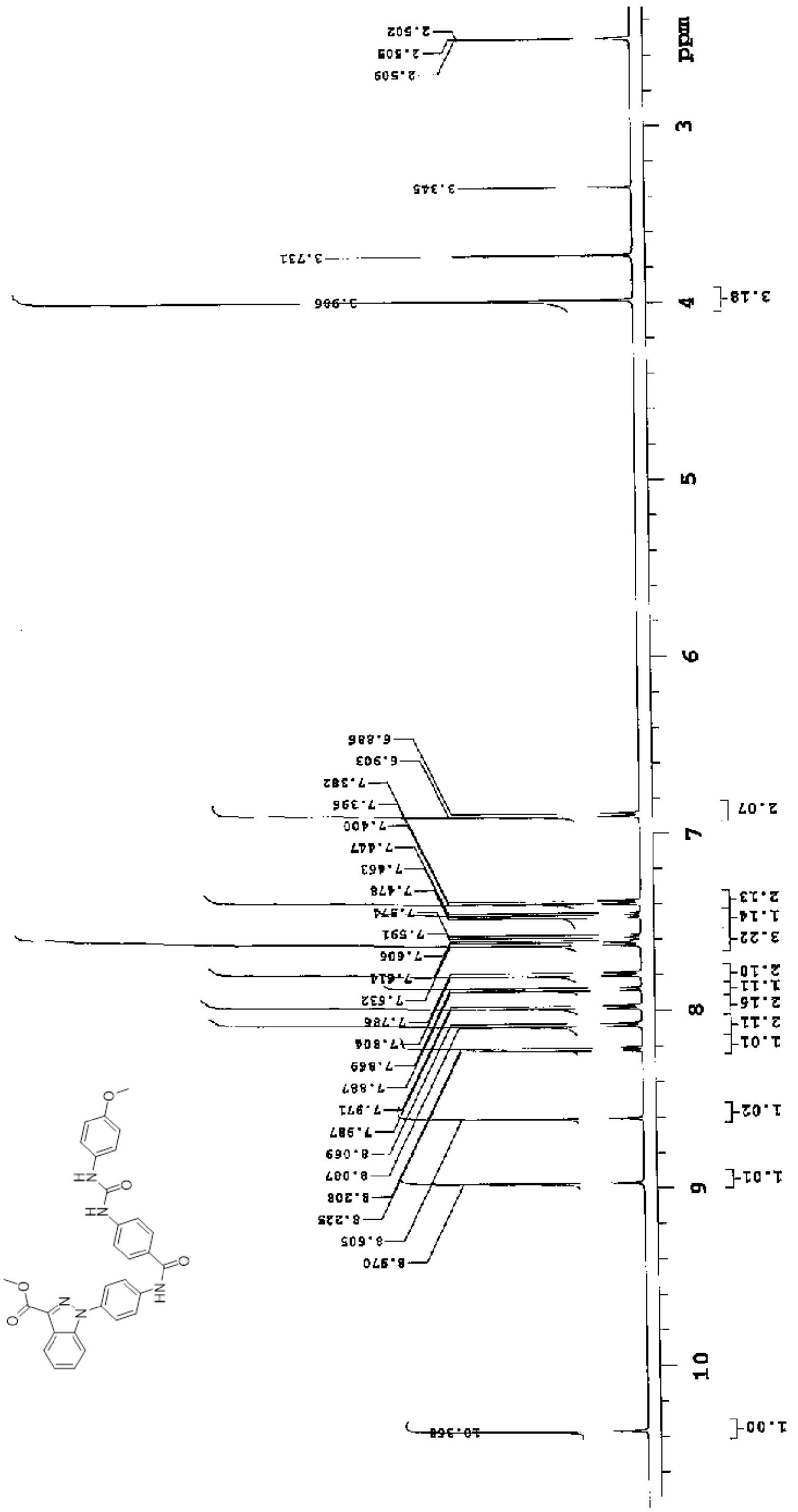

^{13}C NMR: Compound **8e**

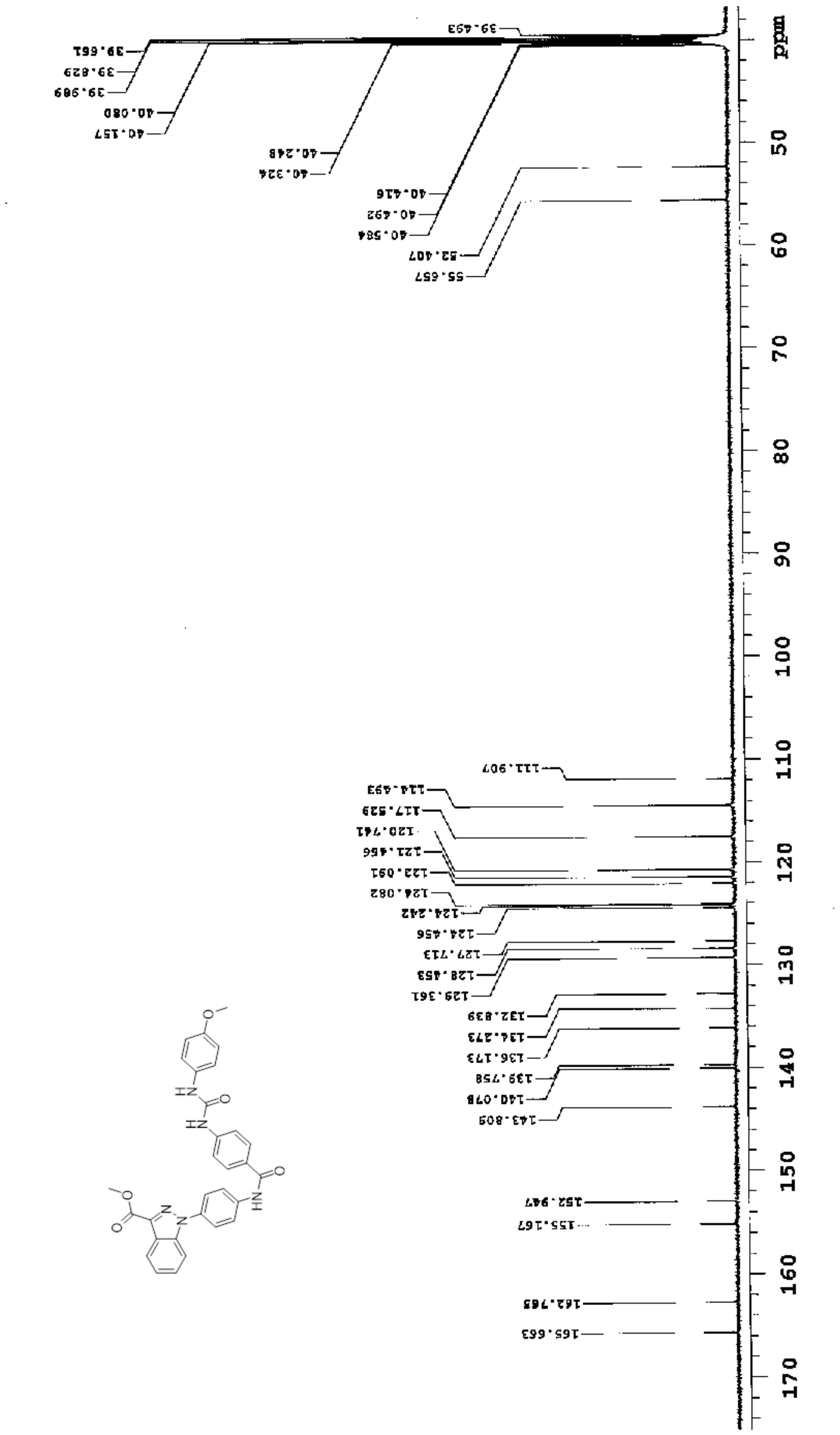

^{1}H NMR: Compound **8f**

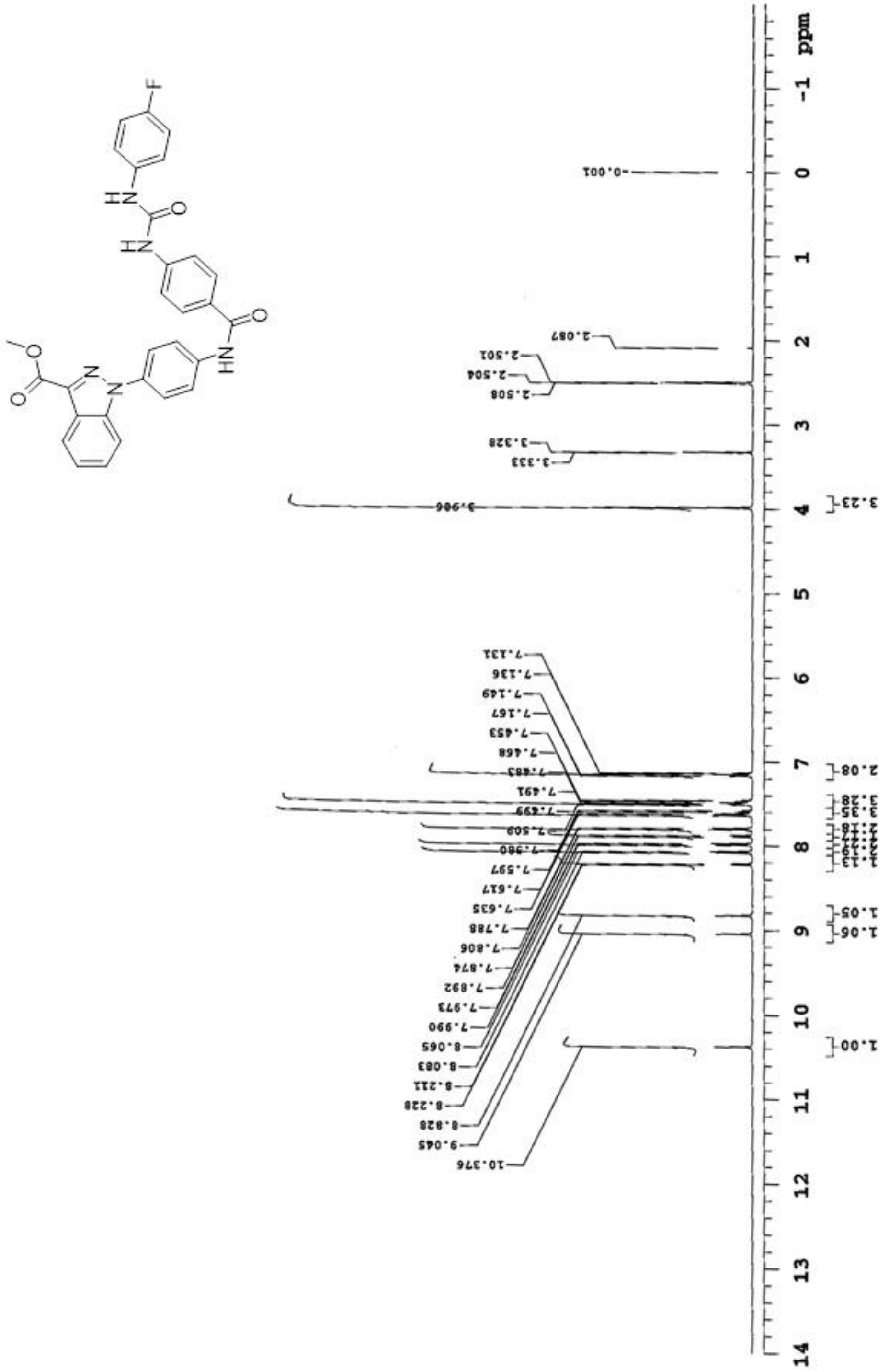

^{13}C NMR: Compound **8f**

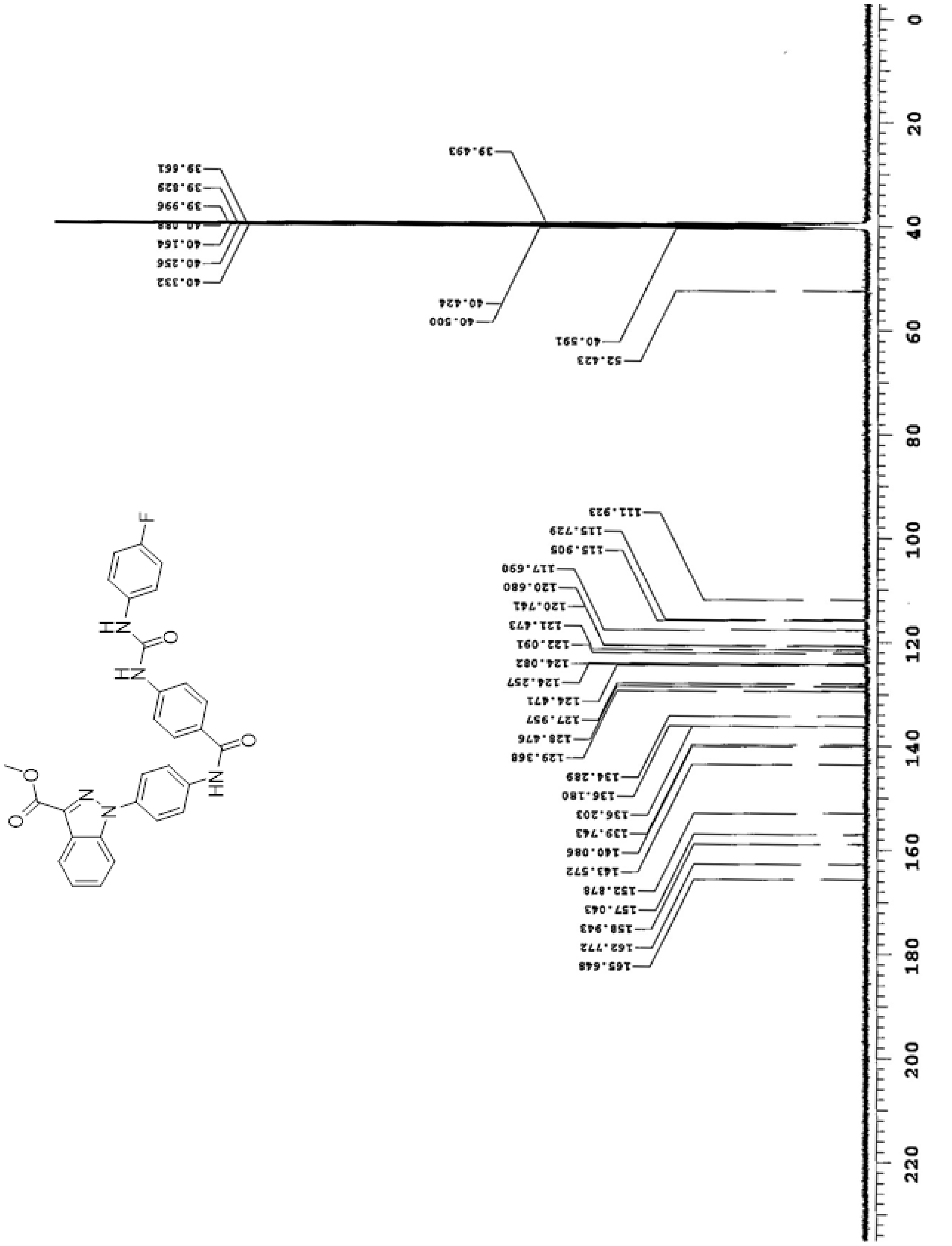

^{1}H NMR: Compound **8g**

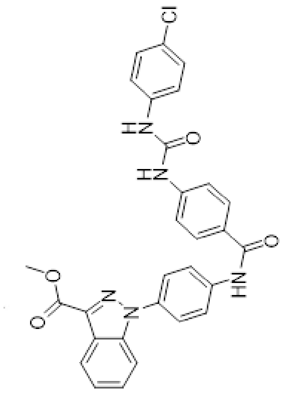

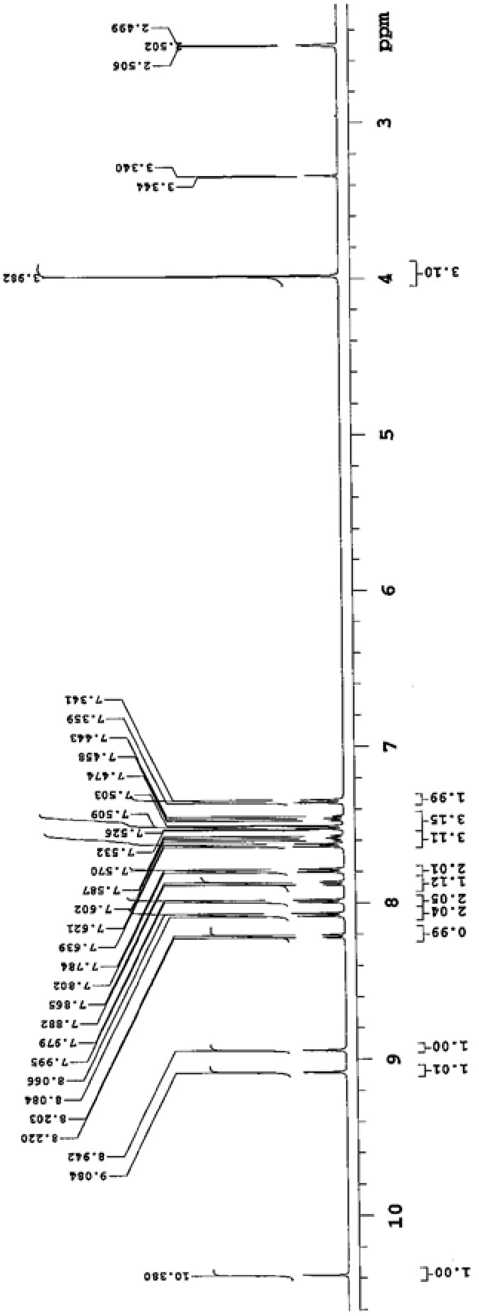

^{13}C NMR: Compound **8g**

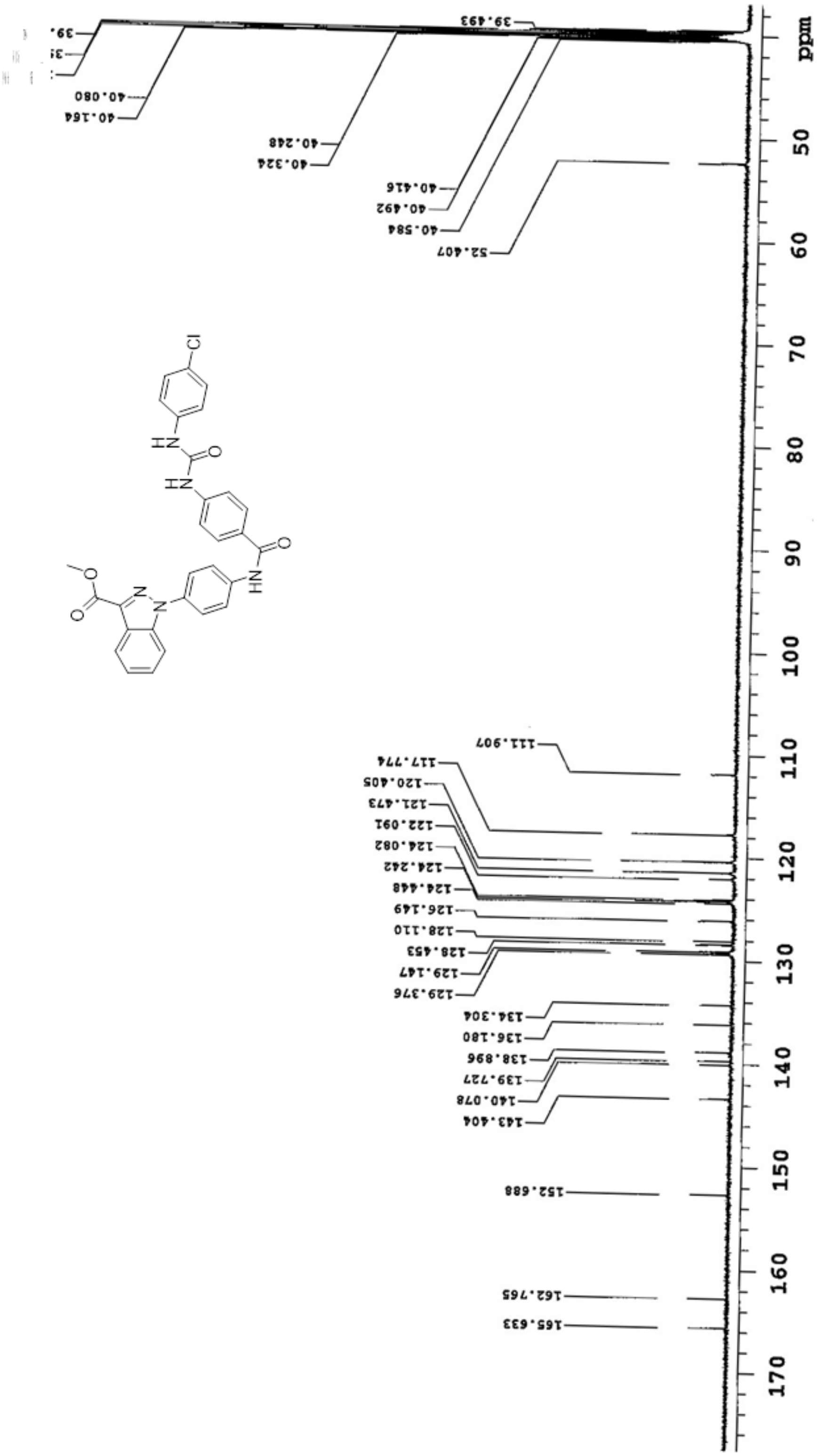

^{1}H NMR: Compound **8h**

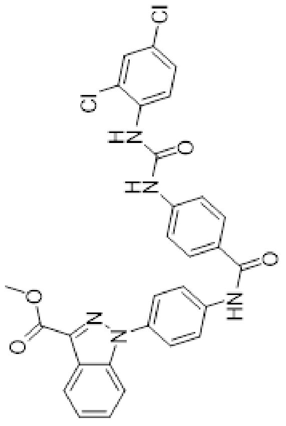

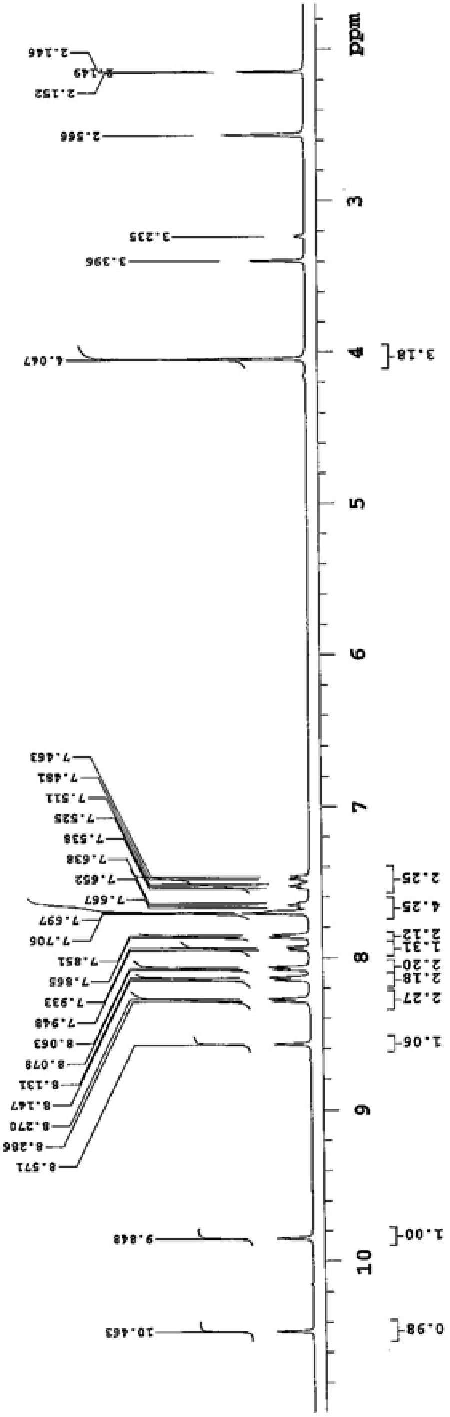

^{13}C NMR: Compound **8h**

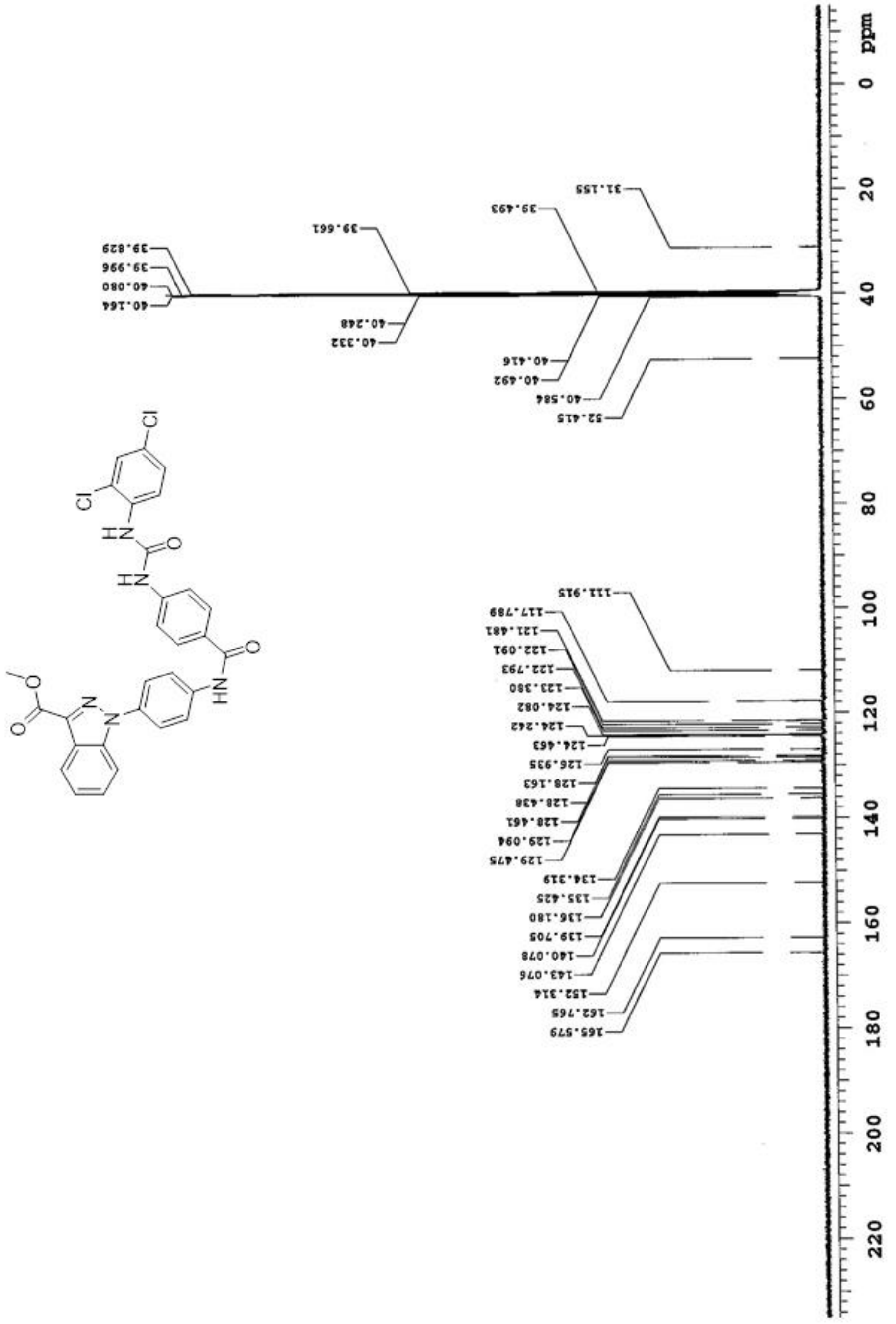

^{1}H NMR: Compound **8i**

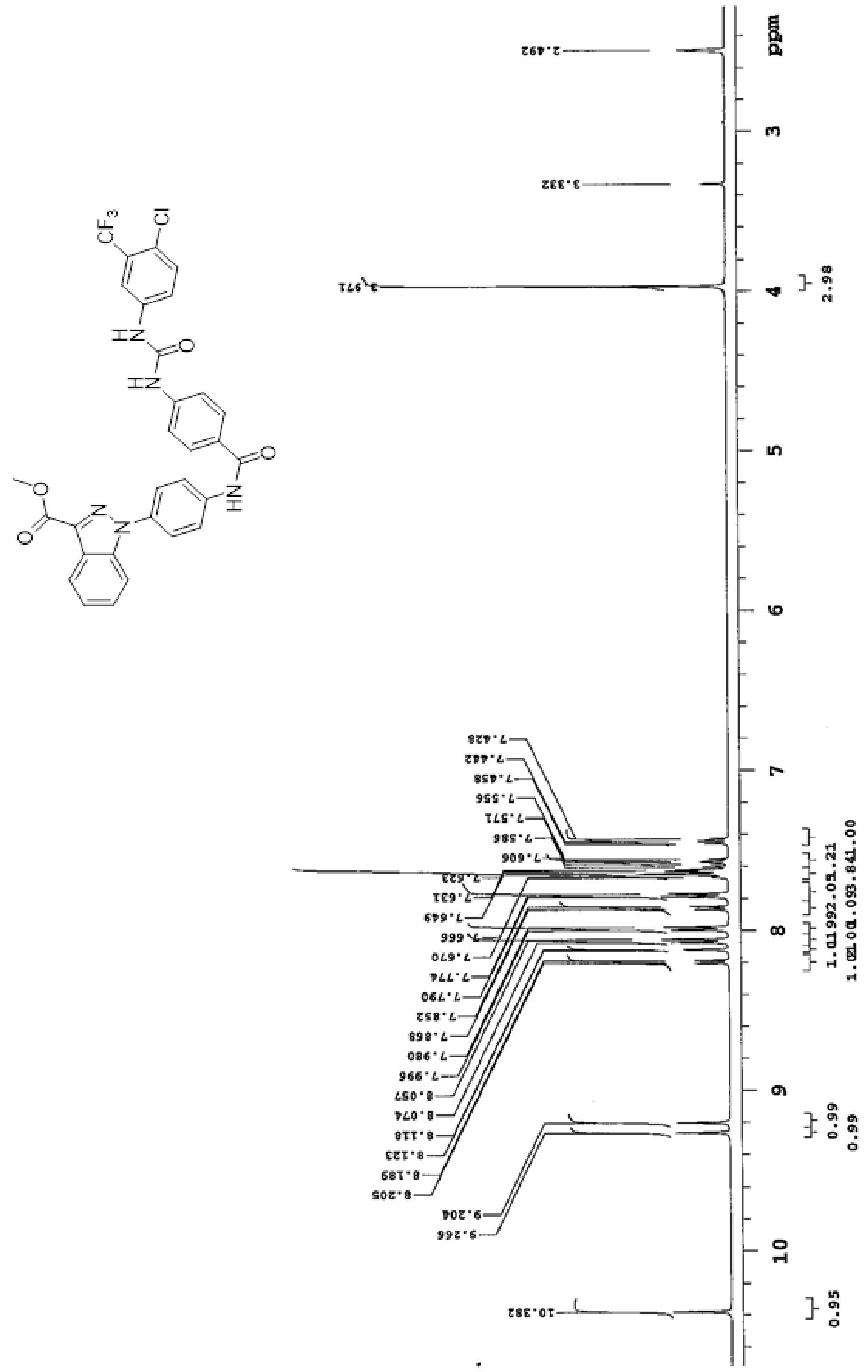

^{13}C NMR: Compound **8i**

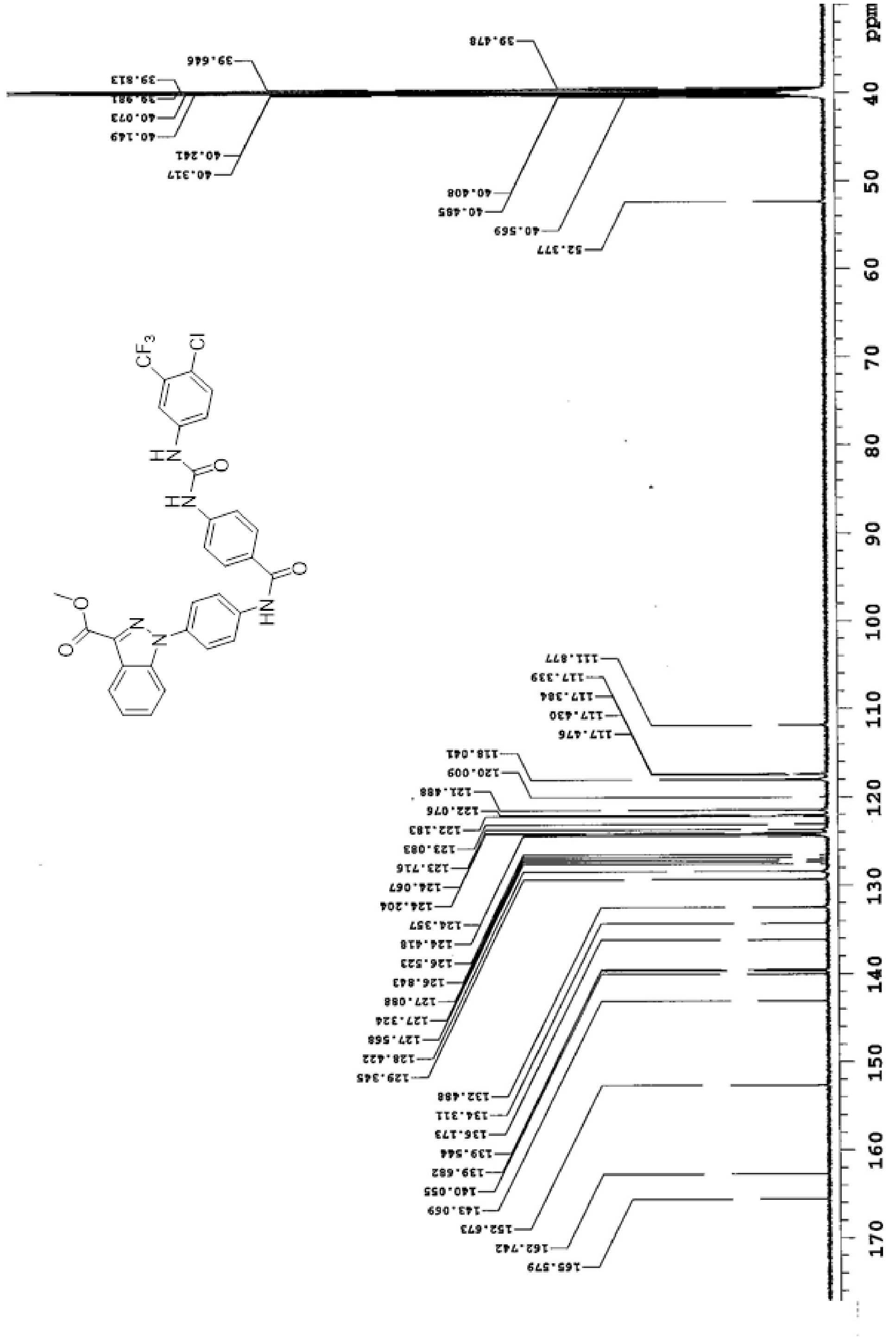

^{1}H NMR: Compound **8j**

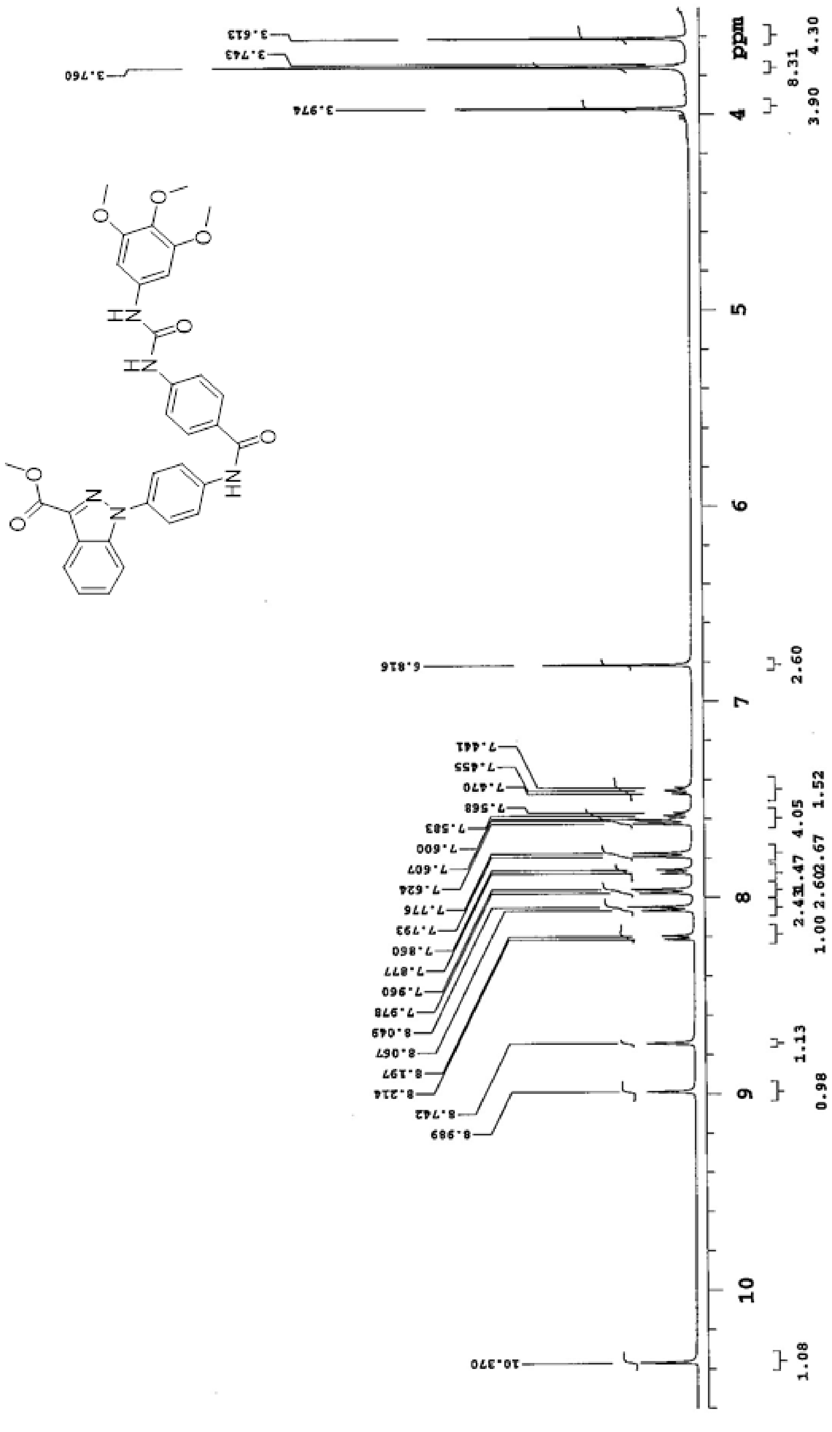

^{13}C NMR: Compound **8j**

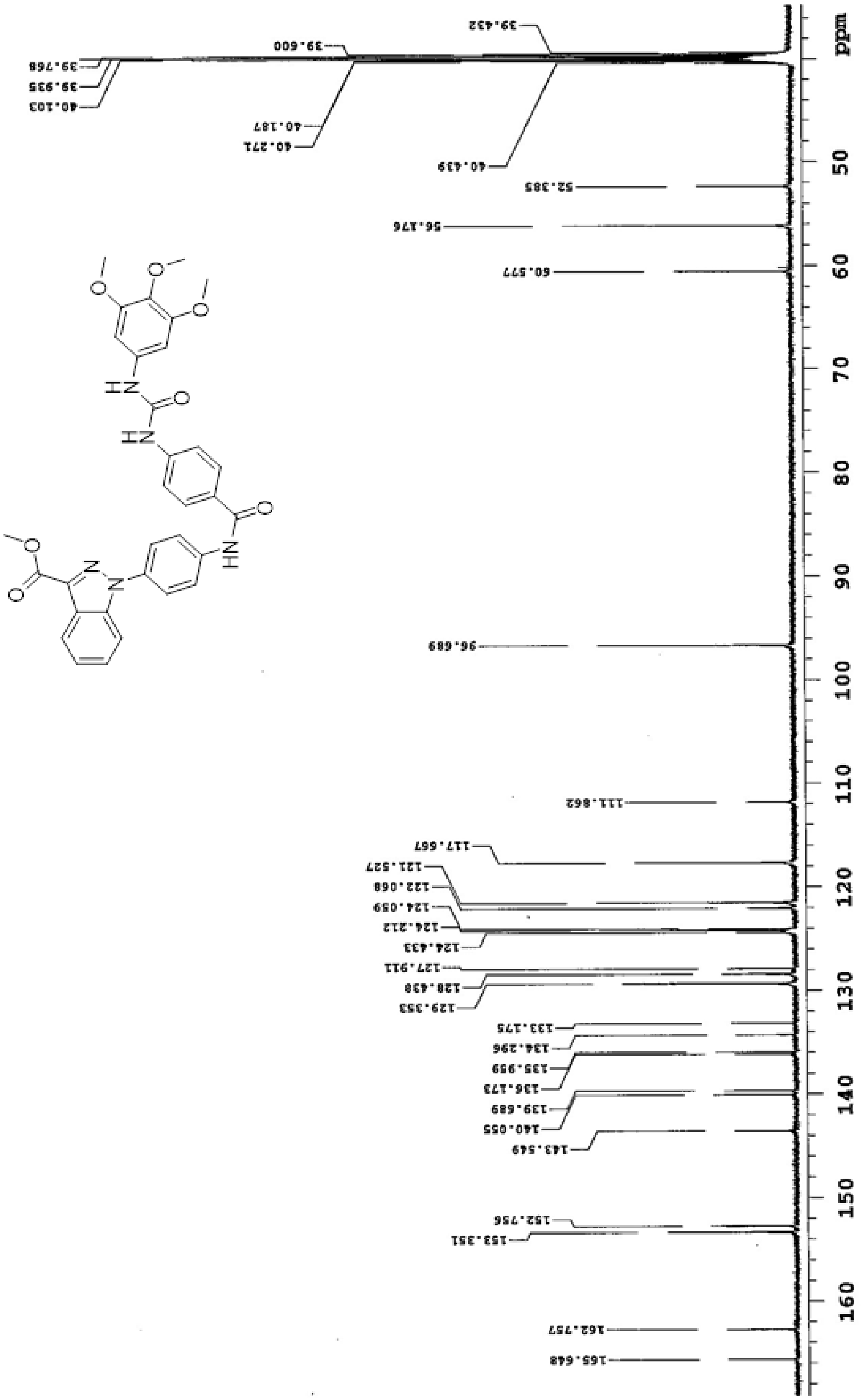

^{1}H NMR: Compound **8k**

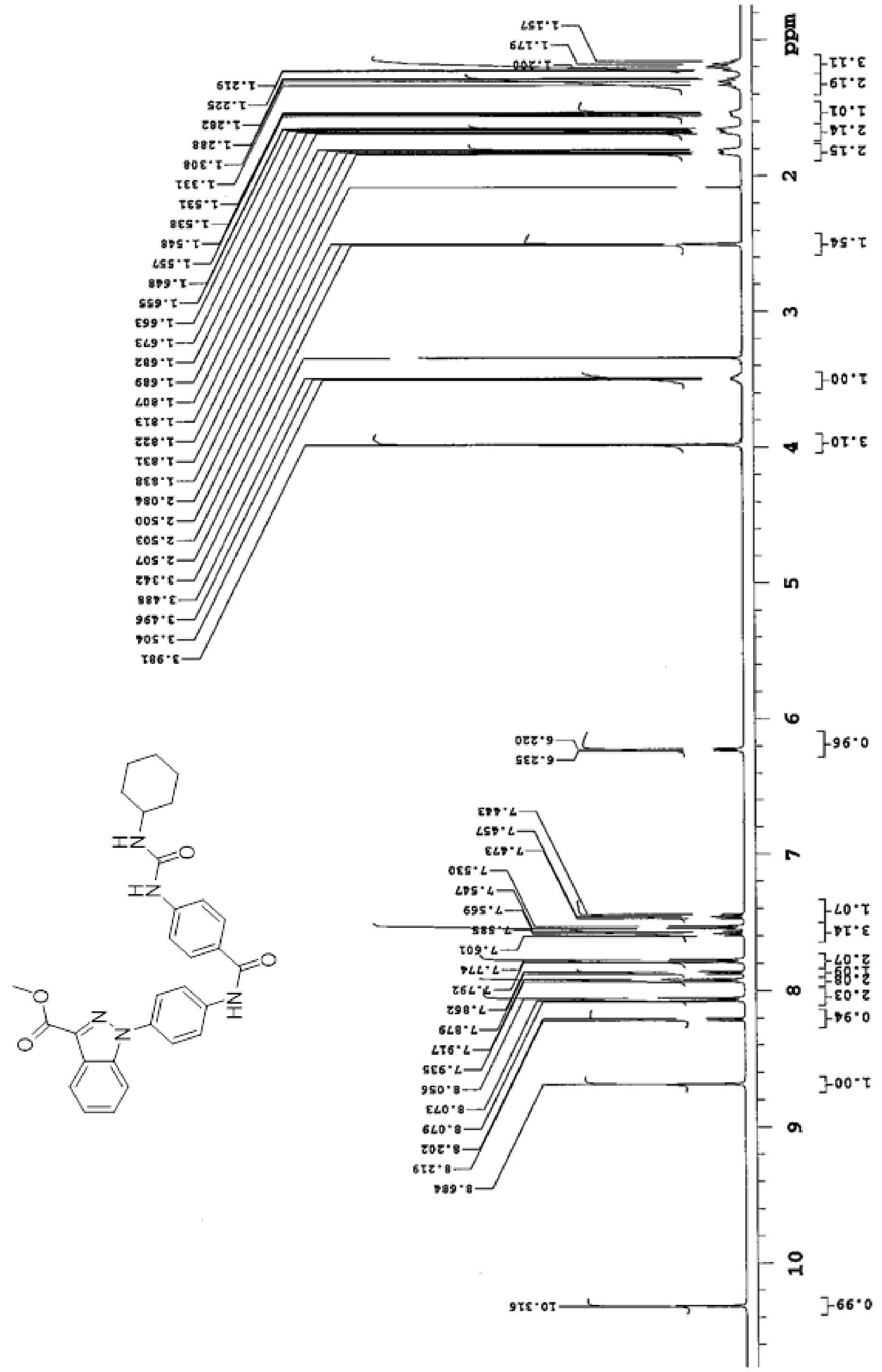

^{13}C NMR: Compound **8k**

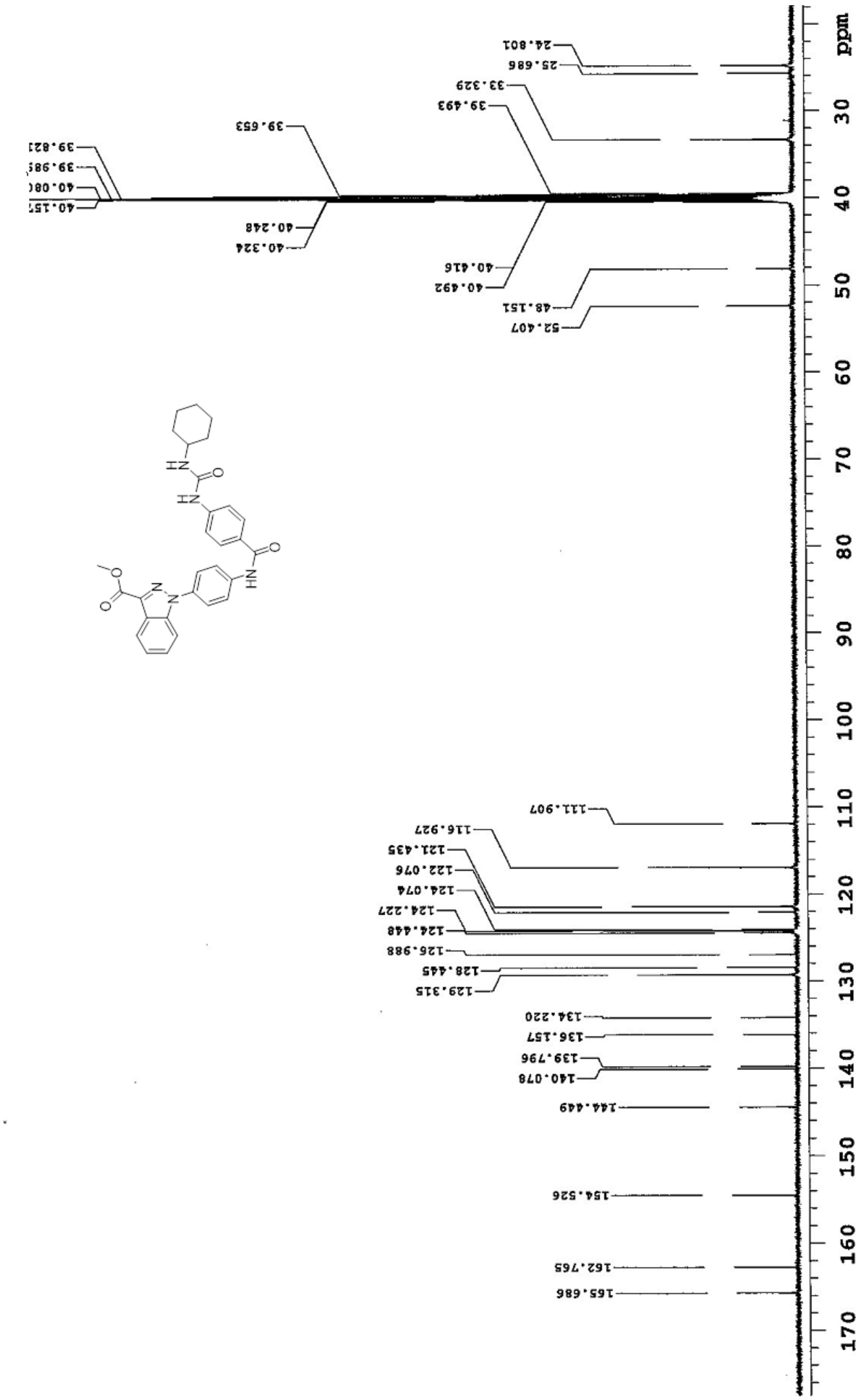

^{1}H NMR: Compound **9**

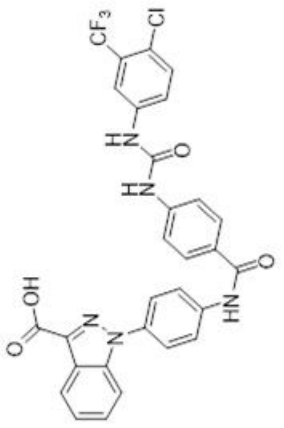

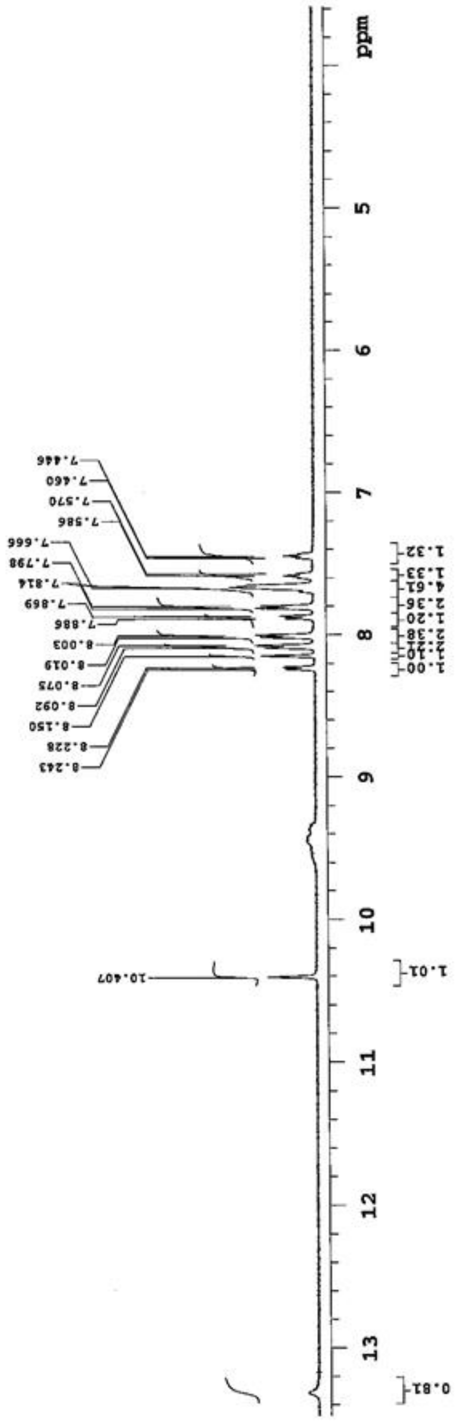

^{13}C NMR: Compound **9**

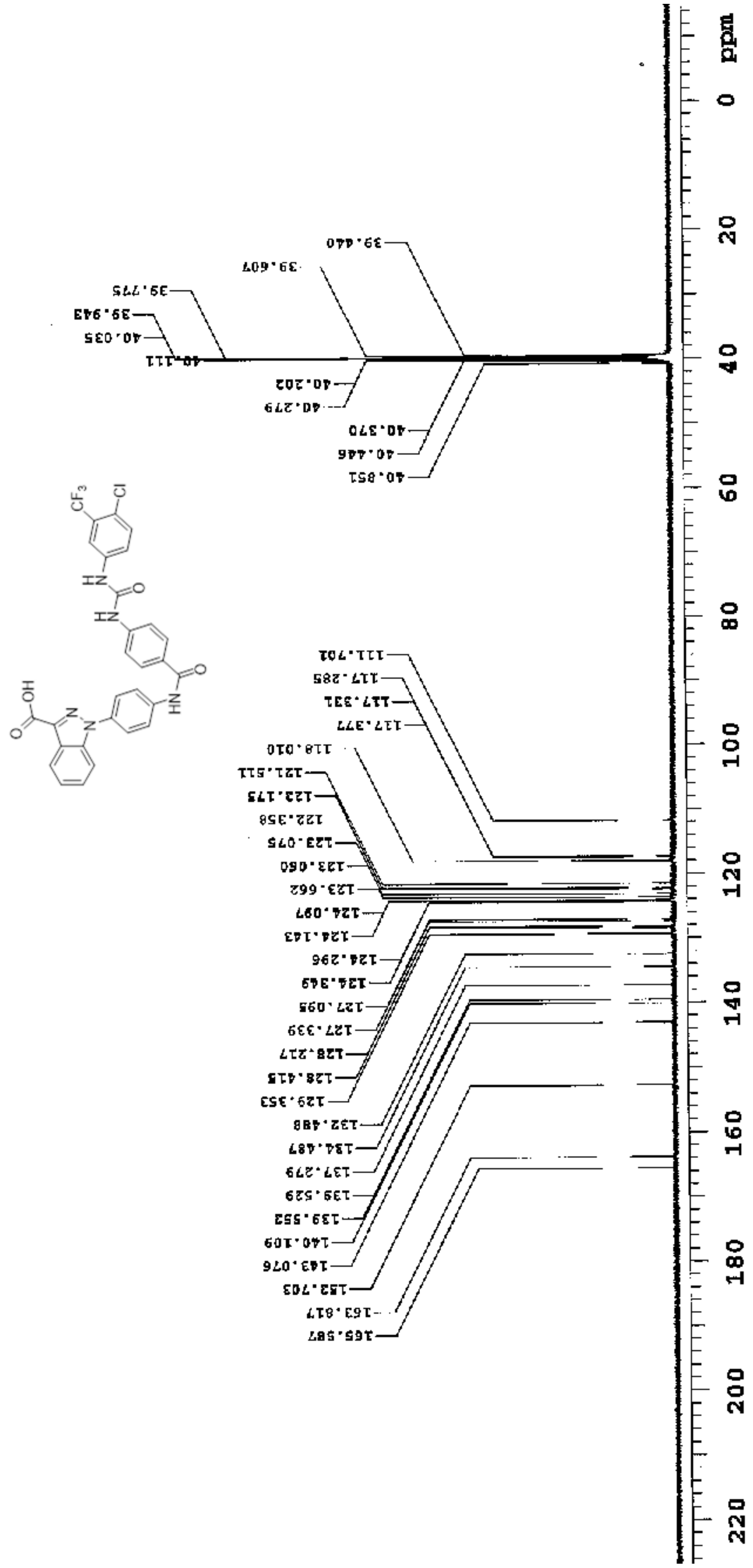

^{1}H NMR: Compound **10**

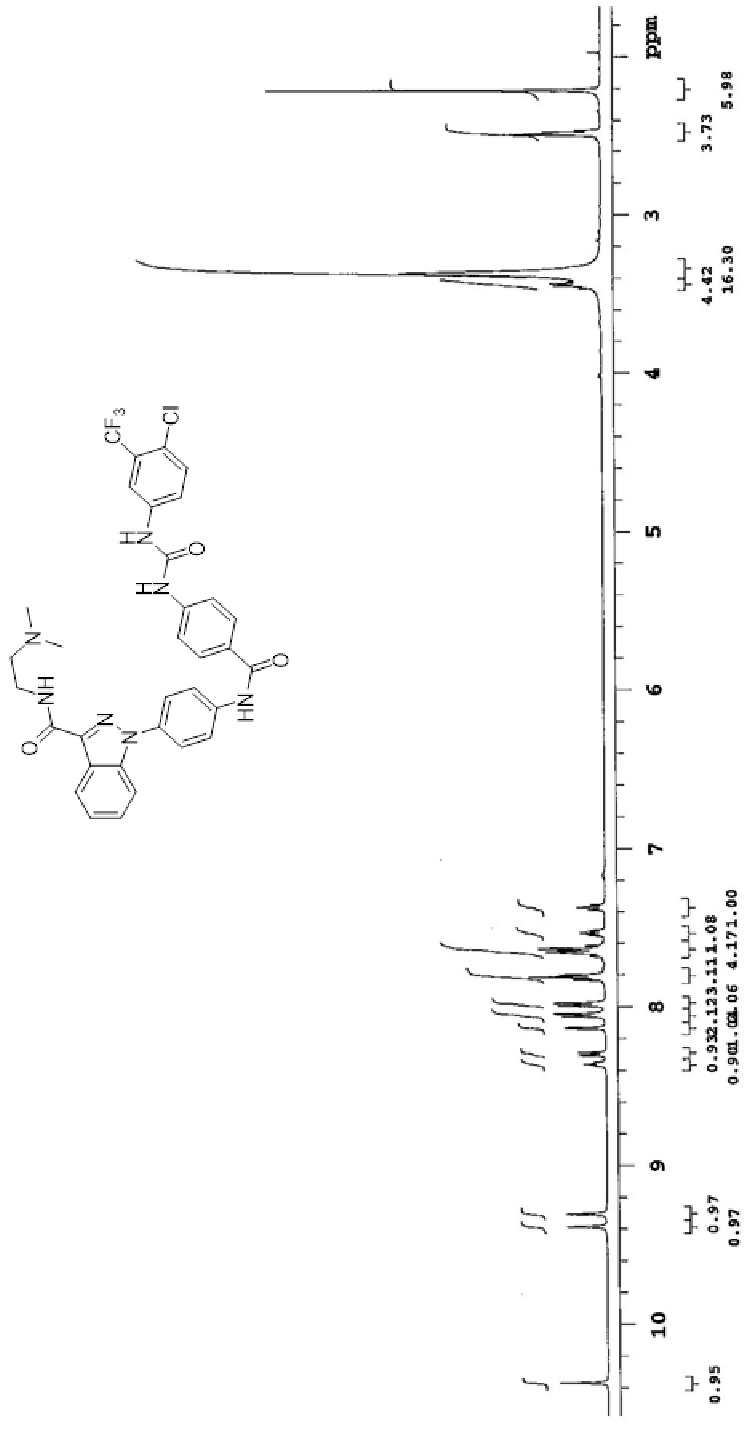

^{13}C NMR: Compound **10**

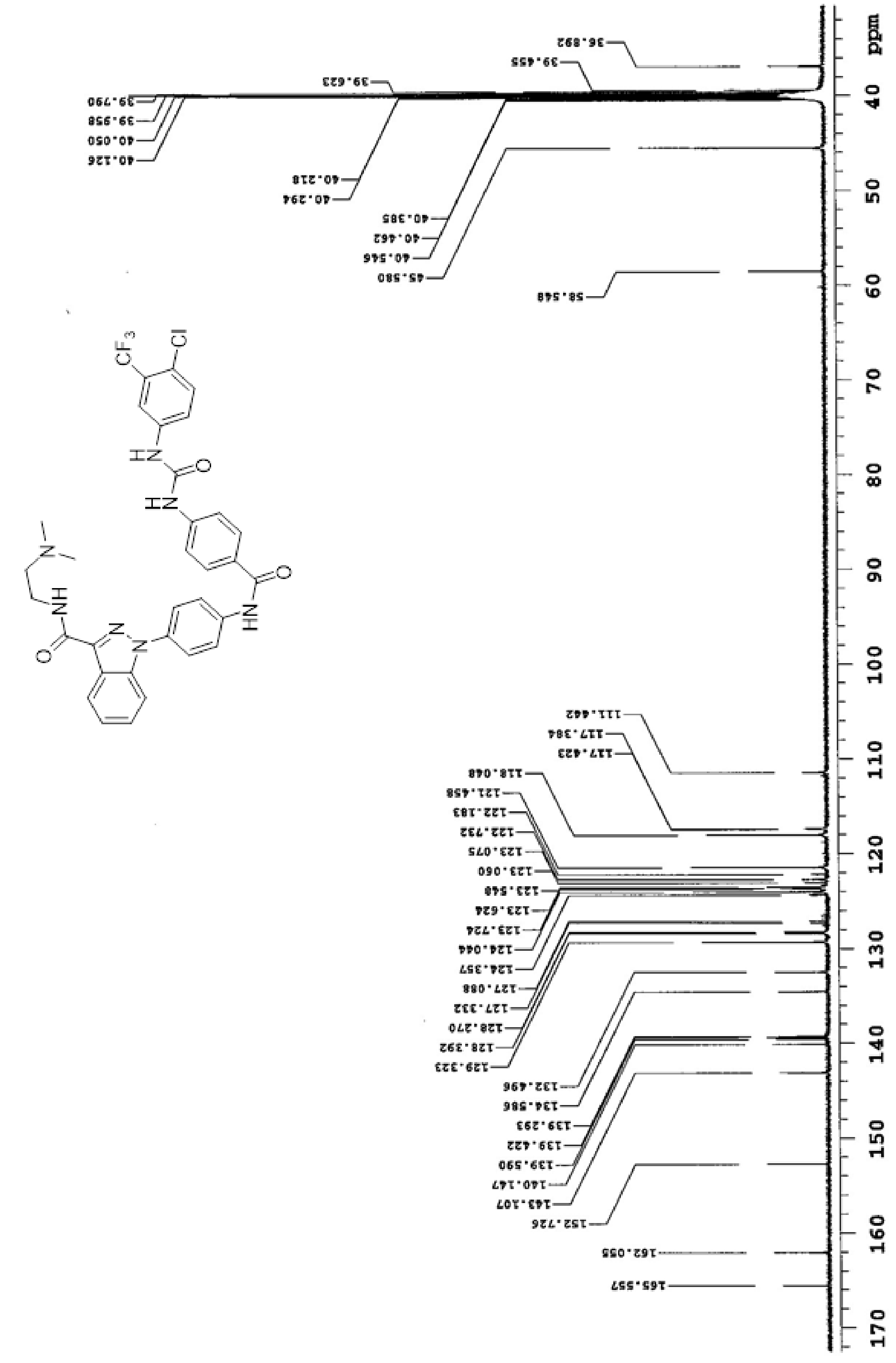

^{1}H NMR: Compound **11**

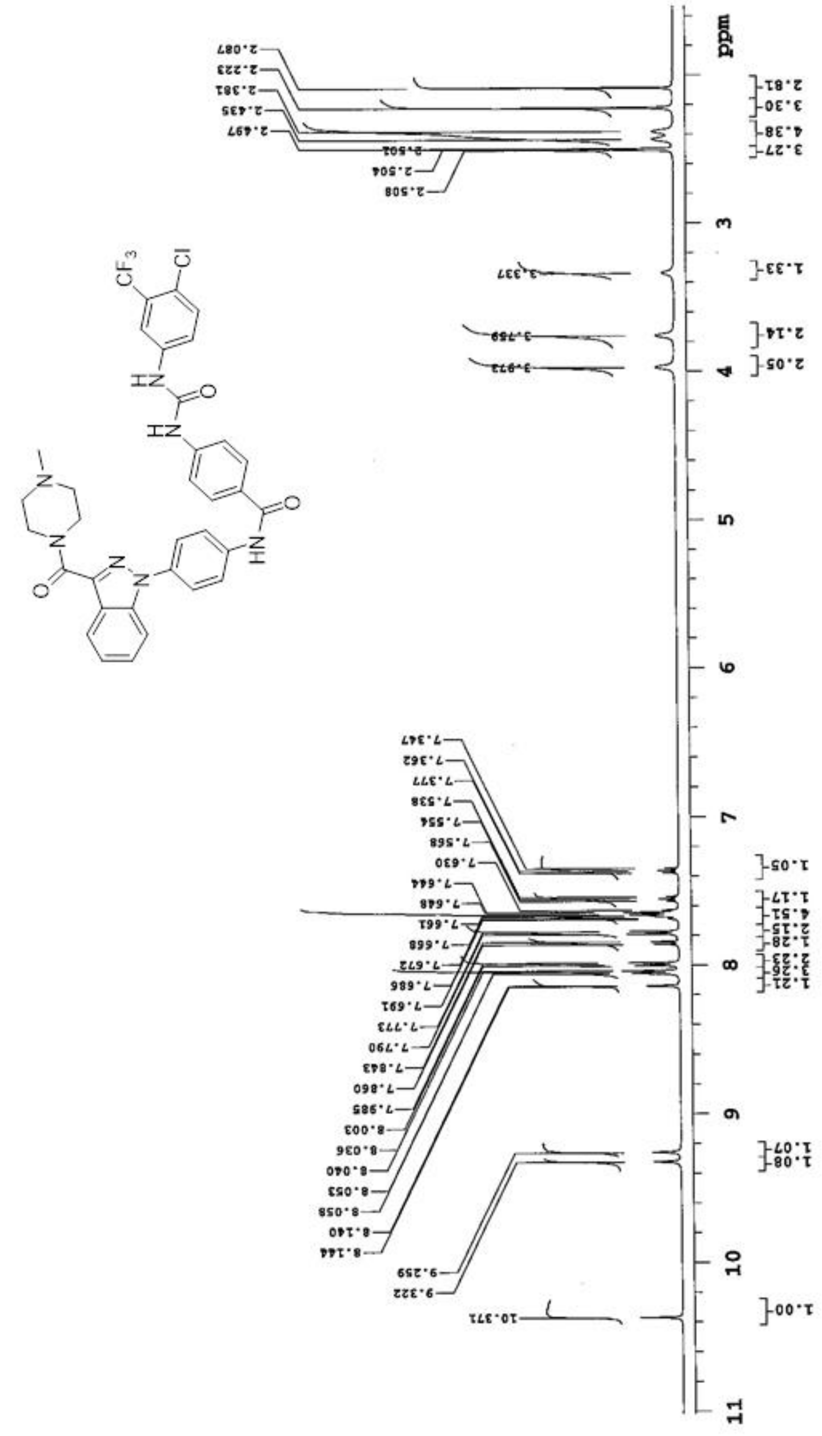

^{13}C NMR: Compound **11**

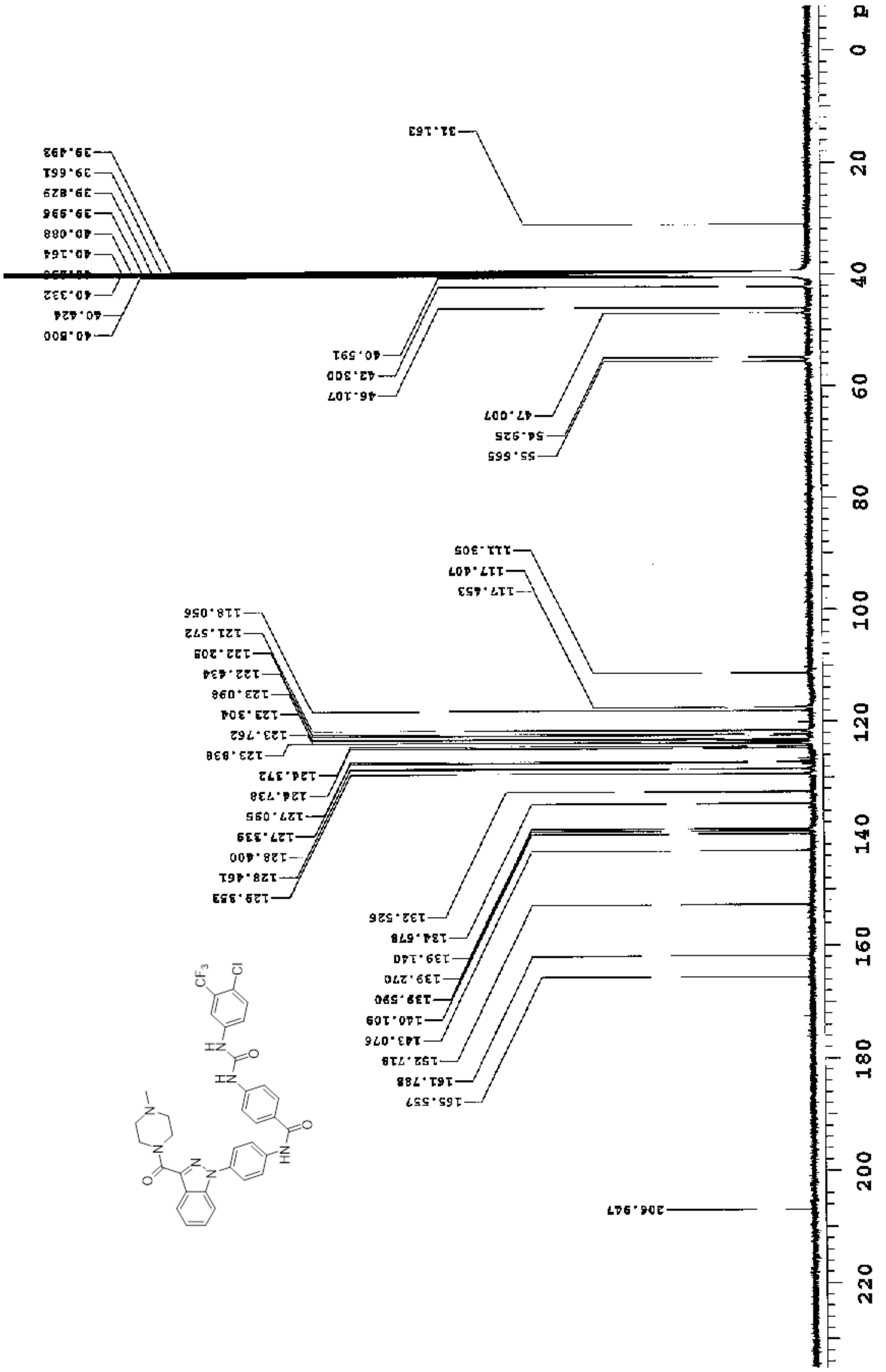

Supplement: Supplementary file 1 — Supplementary Information. [file 41598_2020_74572_MOESM1_ESM.pdf]
